# Local convex hulls for a special class of integer multicommodity flow problems

**DOI:** 10.1007/s10589-016-9831-3

**Published:** 2016-02-12

**Authors:** Zhiyuan Lin, Raymond S. K. Kwan

**Affiliations:** School of Computing, University of Leeds, Leeds, LS2 9JT UK

**Keywords:** Integer multicommodity network flow, Convex hull computation, Rolling stock scheduling

## Abstract

Based on previous work in rolling stock scheduling problems (Alfieri et al. in Transp Sci 40:378–391, 2006; Cacchiani et al. in Math Progr B 124:207–231, 2010; Lin and Kwan in Electron Notes Discret Math 41:165–172, 2013; Schrijver in CWI Q 6:205–217, 1993; Ziarati et al. in Manag Sci 45:1156–1168, 1999), we generalize a local convex hull method for a class of integer multicommodity flow problems, and discuss its feasibility range in high dimensional cases. Suppose a local convex hull can be divided into an up hull, a main hull and a down hull if certain conditions are met, it is shown theoretically that the main hull can only have at most two nonzero facets. The numbers of points in the up and down hull are explored mainly on an empirical basis. The above properties of local convex hulls have led to a slightly modified QuickHull algorithm (the “2-facet QuickHull”) based on the original version proposed by Barber et al. (ACM Trans Math Softw 22:469–483, 1996). As for the feasibility in applying this method to rolling stock scheduling, our empirical experiments show that for the problem instances of ScotRail and Southern Railway, two major train operating companies in the UK, even in the most difficult real-world or artificial conditions (e.g. supposing a train can be served by any of 11 compatible types of self-powered unit), the standard QuickHull (Barber et al. in ACM Trans Math Softw 22:469–483, 1996) can easily compute the relevant convex hulls. For some even more difficult artificial instances that may fall outside the scope of rolling stock scheduling (e.g. a node in a graph can be covered by more than 11 kinds of compatible commodities), there is evidence showing that the “2-facet QuickHull” can be more advantageous over the standard QuickHull for our tested instances. When the number of commodity types is even higher (e.g. >19), or the number of points in a high dimensional space (e.g. 15 dimensions) is not small (e.g. >2000), the local convex hulls cannot be computed either by the standard or the 2-facet QuickHull methods within practical time.

## Introduction

### A special class of multicommodity flow problems

There are several kinds of rolling stock scheduling problems that can be modeled as a class of integer multicommodity flow problems. For instance, the train unit scheduling problem (TUSP) [14, 15], where given a train operator’s timetables and a fleet of train units, an assignment plan has to be determined such that each timetabled train is covered by a single or coupled train units. A notable feature of the TUSP is the unit coupling/decoupling in response to different passenger demands. There are also the train unit circulation problems [2, 12, 21, 23] and the train unit assignment problems [5–8] belonging to this category. The locomotive assignment problem [9, 11, 16, 22, 27, 29] is another kind of rolling stock scheduling problem. A common feature of some of the above problems arising in rolling stock scheduling is the use of computable local convex hulls with respect to each train trip. In this paper, we generalize the use of local convex hull methods for the scenarios found in rolling stock scheduling to a broader spectrum of integer multicommodity flow problems, analyze its feasibility range and pursue more efficient computational approaches, mainly based on instances that are either real-world or artificial from rolling stock scheduling.

Consider an integer multicommodity flow problem [1] defined over a directed acyclic graph ${\mathcal {G}}=({\mathcal {N}},{\mathcal {A}})$ where $j \in {\mathcal {N}}$ and $a \in {\mathcal {A}}$ are the nodes and arcs, and $k \in K$ are the commodities to be flowed in integral amounts from origins to destinations that are specific for each *k*. Each commodity’s total amount may be fixed or bounded by a constant scalar $b_k$. Here we assume that the local constraints are all based on nodes while similar situations on arcs can be derived by analogy. Let $w^j_k\ge 0$ be the flow amount of commodity *k* passing through node *j*. There are compatibility relations between the commodities and the nodes. Let $K_j \subseteq K$ denote the set of commodities that are allowed at node *j*. In general the flow amount for most (if not all) commodities used at a node can take general positive integer values rather than only in $\{0,1\}$, i.e. $w_k^j \in {\mathbb {Z}}_+$.

How the commodities should flow across the network is further restricted by two kinds of local constraints associated with each node and/or arc. The first kind (1) is the provision demand, which requires the total provision achieved at *j* from used commodities to be at least at a required level, as

1 $$\begin{aligned} \sum _{k \in K_j} q_k w_k^j \ge r_j, \quad \forall j \in {\mathcal {N}}, \end{aligned}$$

where $q_k$ is the “task contribution” by one unit of commodity *k* and $r_j$ is the total demand at *j*. The second kind (2) is the bounding restriction, which caps the total resource taken up by used commodities at *j* to be no more than an upper bound, as

2 $$\begin{aligned} \sum _{k \in K_j} v_k w_k^j \le u_j, \quad \forall j \in {\mathcal {N}}, \end{aligned}$$

where $v_k$ is the “resource consumption” taken up by a unit flow amount of commodity *k* and $u_j$ is the total resource upper bound at *j*. We assume, as in many real-world problems, that $q_k,r_j,v_k,u_j$ are integers.

Equations (1) and (2) are commonly seen constraints in integer multicommodity flow problems. To satisfy their requirements, it is sufficient to simply insert them into the associated integer linear program (ILP) after some variable conversion, e.g. $w_k^j=\sum _{p \in {\mathcal {P}}^k_j} x_p$ for a path formulation, where ${\mathcal {P}}^k_j$ is the path set of commodity *k* passing through *j* and $x_p$ is the path variable on path *p* indicating the flow amount along *p*. Therefore, if the variables are based on paths $p \in {\mathcal {P}}^k, k \in K$, and let $c_p$ be the cost of path *p*, the ILP model for a typical integer multicommodity flow problem with the above requirements can be formulated as (*P*): 
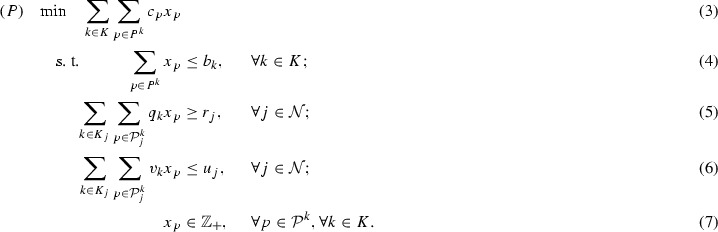


For a TUSP, (*P*) can be interpreted as the following. The nodes (except a source and a sink) in the graph represent the train trips, and the flows of different commodities represent train units of different types that are used to cover the trips. An arc is established between two trips if they can be consecutively served by the same train unit. $b_k$ is the fleet size limit for unit type *k*. A path represents a daily workload for a train unit vehicle as a sequence of train trips and $x_p$ is the number of train units used for that sequence. In addition, $q_k$ is the number of seats (capacity) in a train unit of type *k* and $r_j$ is the passenger demand measured in number of seats for train *j*, while $v_k$ is the number of cars of a unit of type *k* and $u_j$ is the maximum number of cars for coupled unit formations that platforms can accommodate regarding trip *j*. The objective minimizes the total cost of deployed units. See [6, 14] and [15] for further details. As a generic integer multicommodity flow ILP formulation, (*P*) or its variants can also be interpreted as many other real-world problems[1].

As a standard integer multicommodity flow formulation, there are certain disadvantages or incapabilities in directly using (1) and (2) (or (5) and (6) as in (*P*)).

(i) *Weak linear programming (LP) relaxation * As often observed, the two kinds of constraints are very likely to yield weak LP relaxation due to their knapsack nature. The train unit/locomotive scheduling (assignment, circulation) problems are typical examples where the $q_k,r_j$ are measured in number of passengers and $v_k,u_j$ are measured in number of cars.
(ii) *Combination-specific upper bounds * The bounding restriction may be combination-specific, i.e. the upper bound will vary according to different combinations in terms of which and how many commodities are used. The complex upper bound restrictions for coupled train units in the train unit scheduling problem [14, 15] is such an example. If different constraints with different bounds are formed, *disjunctive* relations among these constraints may be required. However coexisting constraints in an LP are *conjunctive* thus will not realize the target. One remedy would be to introduce extra binary variables and constraints to represent the use of different combinations, as given in [15]. This however may often slow down the solution process.
(iii) *Commodity compatibility * Sometimes there can be compatibility relations among the commodities used at a node where only certain collections of them can coexist. For instance, when five commodities $k_1,\ldots ,k_5$ are allowed at a node, not just any of them can be used together. A rule may require such that one can only use a combination from $k_1$ and $k_2$ or a combination from $k_3,k_4,k_5$ but nothing else. Therefore, if flows from both $k_1$ and $k_3$ are used at the node, it should be deemed as invalid. The creation of train unit families based on the train unit coupling compatibility introduced in [14, 15] is a result of commodity compatibility relations. Take the above $k_1,\ldots ,k_5$ as an example. $k_1$ and $k_2$ can represent diesel train units while $k_3,k_4$ and $k_5$ can be electric train units. A unit type from the diesel family cannot be coupled with a unit from the electric family.

To deal with the weak LP relaxation problem above, a class of similar methods has been proposed in several papers [2, 6, 23, 29], which happen to be all in railway rolling stock assignment/scheduling problems. Also in dealing with all the three points listed above, a method for directly computing “train convex hulls” has been given in [14]. We will refer to this class of method as the *local convex hull method* and will generalize it in the subsequent sections. Admittedly this method will not be universal for all integer multicommodity flow problems. To apply it successfully, a problem should possess certain features.

#### **Observation 1**

For the nodes $j \in {\mathcal {N}}$, the number of commodities suitable for node *j*, i.e. $|K_j|$, should not be “large”, although the total number of commodities over the network may still be “large”. For the commodities suitable at node *j*, the number of valid commodity combinations should also not be “large”. Finally, the flow amount of each commodity at *j* should be able to take appropriate general nonnegative integer values, i.e. $w_k^j \in {\mathbb {Z}}_+$, rather than only binary, i.e. $w_k^j \in \{0,1\}$.

The meaning of “large” in Observation 1 can be problem-specific. In the train unit scheduling instance we tested, it can be regarded as “large” when $|K_j|>19$ and/or the number of valid commodity combinations is above 1200.

The features in Observation 1 are commonly seen in a class of integer multicommodity flow problems, typically arising in rolling stock scheduling problems as train unit or locomotive scheduling, where the nodes are train services to be covered by rolling stock, the commodities are different types of rolling stock and $w_k^j$ are the number of rolling stock of type *k* used for train *j*. Despite the above features, those scheduling problems are generally very difficult to solve as the number of nodes can be from hundreds to several thousands with a very high density of arcs.

In addition, there are also other real-world applications formulated as integer multicommodity flow problems having the features mentioned in Observation 1 and are thus suitable for the local convex hull method. Note that their specific formulations do not have to be exactly the same as (*P*). One example is the Multivehicle Tanker Scheduling Problem (Bellmore and Bennington [4]), which can be formulated as an integer multicommodity flow problem in maximizing the total utilities achieved by a fleet of heterogeneous tankers to meet a prescribed schedule of deliveries. In its corresponding network, an arc represents a shipment that can be shared by different types of tanker and is upper bounded due to limited delivery location capacity while dissimilar types differ in carrying capabilities and other factors. The arc-based type-specific flow variables represent the number of deployed tankers for the shipments of corresponding arcs and can take integer values other than binary. The number of tanker types in the fleet will be generally not very large, also following Observation 1. Moreover, some problems that are not categorized as integer multicommodity flow types also satisfy Observation 1. The Generalized Transportation Problem (Wolsey [28]) is such an example, where the demands of clients have to be satisfied by trucks of different types that can be used together for the same client. Notably, the number of trucks of the same type deployed for the same client can take non-binary positive integers.

Finally the structures of the local convex hulls may differ as the formulations differ. For example, in most rolling stock scheduling problems, since there are two kinds of bundle constraints (5 and 6), the main hull to be introduced in Sect. 2 has at most two nonzero facets. However, for the Multivehicle Tanker Scheduling Problem [4], there is no constraint of type (6). Thus the main hull only has one and only one nonzero facets. These differences will not prevent the using of the generalized local convex hull method and the customized convex hull computation algorithms in Sects. 2 and 3.

Here we propose the local convex hull method and its relevant convex hull computation algorithms based on the train unit scheduling problem [14, 15]. Their applications to other suitable problems can be derived by analogy.

### Local convex hull method

A generalization on the local convex hull methods arising in rolling stock scheduling to a generic integer multicommodity flow problem is given here. Here each local convex hull corresponds to a single node in the network where Constraints (5) and/or (6) are applied. For each node *j*, a commodity combination set $W_j$ is defined as

8 $$\begin{aligned} W_j=\bigg \{w^j\in {\mathbb {Z}}_+^{K_j}\bigg |\forall w^j: \text {a valid commodity combination for node } j \bigg \}, \quad \forall j \in {\mathcal {N}},\nonumber \\ \end{aligned}$$

where $w^j=(w^j_1,\ldots ,w^j_{|K_j|})^T$ is a vector representing the flow amounts of a commodity combination. We assume that due to Observation 1 the number of combinations are small enough such that $W_j$ can be simply obtained by enumeration. For problem instances with the demand and bounding restrictions exactly given by (1) and (2), we also have

9 $$\begin{aligned} W_j=\Bigg \{w^j\in {\mathbb {Z}}_+^{K_j}\Bigg |\displaystyle \sum _{k \in K_j} q_k w_k^j \ge r_j, \sum _{k \in K_j} v_k w_k^j \le u_j\Bigg \}, \quad \forall j \in {\mathcal {N}}. \end{aligned}$$

However, for cases with combination-specific upper bounds and commodity compatibility relations, the combination set may only be obtained from (8) by enumeration. Next for each node the *local convex hull* ${\mathrm {conv}}(W_j)$ of the above combination set is computed explicitly before the optimization process, given that the number of points $|W_j|$ is not too large and the dimension $|K_j|$ is appropriately small:

10 $$\begin{aligned} {\mathrm {conv}}( W _j)=\bigg \{w^j\in {\mathbb {R}}_+^{K_j}\bigg |H^j w^j \le h^j \bigg \}, \quad \forall j\in {\mathcal {N}}. \end{aligned}$$

The local convex hull (10) is described by nonzero facets $f \in F_j$ such that $H^j \in {\mathbb {R}}^{F_j \times K_j}$ and $h^j \in {\mathbb {R}}^{F_j}$. Via variable conversion $w_k^j=\sum _{p \in {\mathcal {P}}_j^k}x_p$, the demand and upper bounding requirements at node *j* can be satisfied by the following local convex hull constraints

11 $$\begin{aligned} \sum _{k \in K_j} \sum _{p \in P_j^k} H_{f,k}^j x_p \le h_f^j, \forall f \in F_j, \quad \forall j\in {\mathcal {N}}. \end{aligned}$$

Here $H_{f,k}^j$ is the entry corresponding to commodity *k* in facet *f* of $H^j$; $h_f^j$ is the entry corresponding to facet *f* in $h^j$. $H_{f,k}^j$ and $h_f^j$ can be either positive or negative. Now by replacing (5) and (6) in (*P*) with (11), we have $(P^\prime )$, the integer multicommodity formulation with local convex hulls: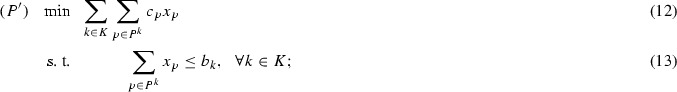 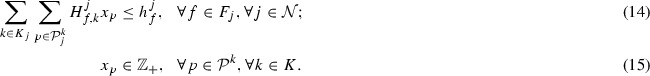


Note that the upper bounds requirements that are combination-specific can be automatically satisfied by (14) as long as they can be described by a set of linear inequalities. One of the most beneficial effect from using the convex hull constraints (14) is that often a much tightened LP relaxation will be obtained compared with solely using (5) and (6). In addition, this pre-processing on local convex hull computation is carried out before solving the ILP and thus will not yield additional burden to the solution process on the ILP itself.

#### Removing incompatible commodities

The aforementioned point (iii) shows that logical non-linear restrictions on commodity compatibility can occur. Generally, the local convex hulls can remove part of the incompatible commodities but not all of them. Therefore, subsequent extra methods in ensuring all jointly used commodities at each node are compatible have to be designed and applied. We use an example to illustrate this.

**Fig. 1:**
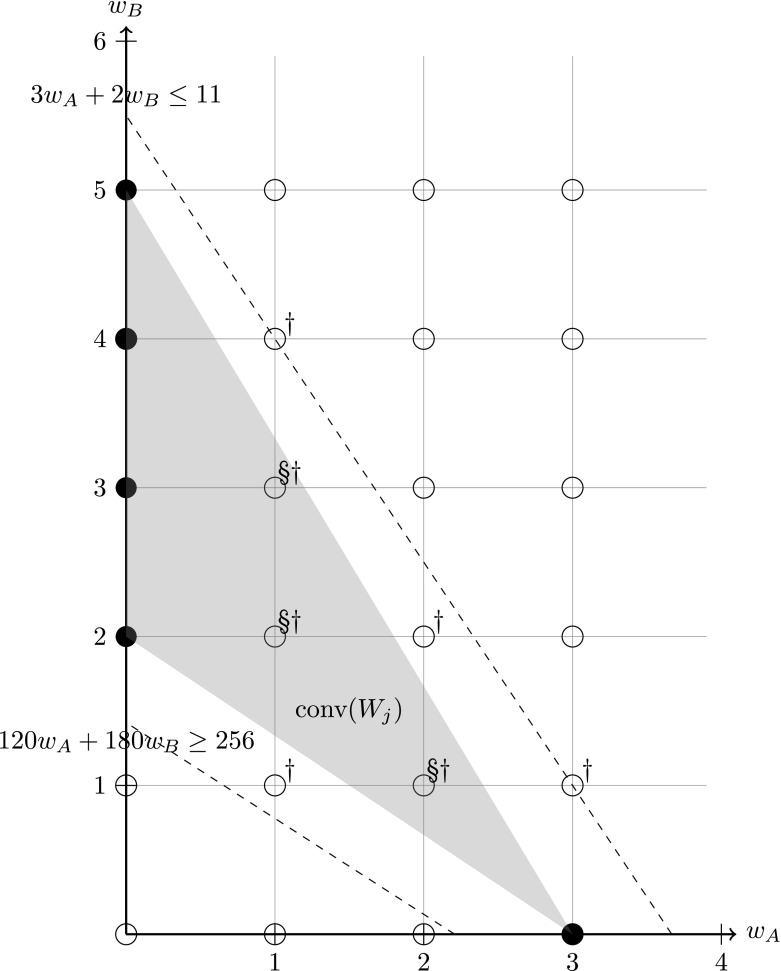
Integer points from incompatible commodities *A* and *B* at *j*

Suppose two incompatible commodities *A* and *B* are permitted at node *j*, whose demand is $r_j=256$ and an upper bound $u_j=11$ is imposed on both *A* and *B*. The task contribution $q_k$ and resource consumption $v_k$ of *A* and *B* are given in Table 1. Since *A* and *B* are incompatible, the valid points are all on the two axes: $W_j=\{(3,0), (0,2), (0,3), (0,4), (0,5)\}$, and their convex hull ${\mathrm {conv}}(W_j)$ can be computed. Figure 1 illustrates the integer points included in the solution space in the LP relaxation by either directly using (1) and (2) ($3w_A+2w_B \le 11$ and $120w_A+180w_B \ge 256$), or using the convex hull ${\mathrm {conv}}(W_j)$. The filled integer points are valid combinations while the blank integer points are invalid. Because of the nature of linear programming constraints, the invalid points due to having both the commodities cannot be eliminated either by the direct constraints (such points are labeled “†”) or the convex hull (“§”). Nevertheless, from Fig. 1, it can be observed that since the convex hull has narrowed down the solution space, it has already removed four invalid points (†) compared with direct constraints. On the other hand, for the remaining three points (§†), it is important to have a method that can further remove them.

**Table 1:** Two incompatible commodities to cover a node *j* with $r_j=256,u_j=11$

Commodities *k*	$q_k$	$v_k$
*A*	120	3
*B*	180	2

Here we give an example on such a method named “train-family branching” proposed in [14]. Define compatible commodities to be in the same *family*. Each time after a relaxation is solved at a BB tree node $n_{{\mathrm {BB}}}$, this branching scheme identifies a graph node $j \in {\mathcal {N}}$ covered by multiple families $\varphi _1,\ldots ,\varphi _n$. Let $\varPhi _j$ be the set of all families allowed at *j*. Then $n+1$ (or *n*) branches are formed at $n_{{\mathrm {BB}}}$:

- For the first 1...*n* branches, say at the *i*-th branch where $i \in \{1,\ldots ,n\}$, only commodities from family $\varphi _i$ will be allowed to cover *j*.
- For the last $(n+1)$-th branch, if $\varPhi _j \backslash \{\varphi _1,\ldots ,\varphi _n\} \ne \emptyset$, then commodities from families $\varphi _1,\ldots ,\varphi _n$ will be forbidden to cover *j*; if $\varPhi _j \backslash \{\varphi _1,\ldots ,\varphi _n\} = \emptyset$, then the $(n+1)$-th branch is not needed.

The above branching scheme can be easily implemented by deleting certain columns in the restricted master problem and certain arcs in the subproblem network if a branch-and-price is used to solve $(P')$.

#### A real-world example from Southern Railway

We use a real-world example from the fleet of Southern Railway, UK to illustrate the above convex hull preprocessing approach. Table 2 gives the possible combinations and coupling upper bounds for two compatible unit types of c455/8 and c456/0 from Southern Railway. There are two kinds of coupling upper bounds measured in the number of cars and units respectively. In practice, the number of restrictive factors may be larger than two, giving more complicated restrictions that are unable to be represented by linear constraints.

**Table 2:** Combination-specific coupling upper bounds (UB) for unit type c455/8 and c456/0

Combination	UB in car#	UB in unit#
c455/8 (4-car)	8 (2$\times$c455/8)	2
c456/0 (2-car)	6 (3$\times$c456/0)	3
Mixed	8 (c455/8+2$\times$c456/0)	3

**Fig. 2:**
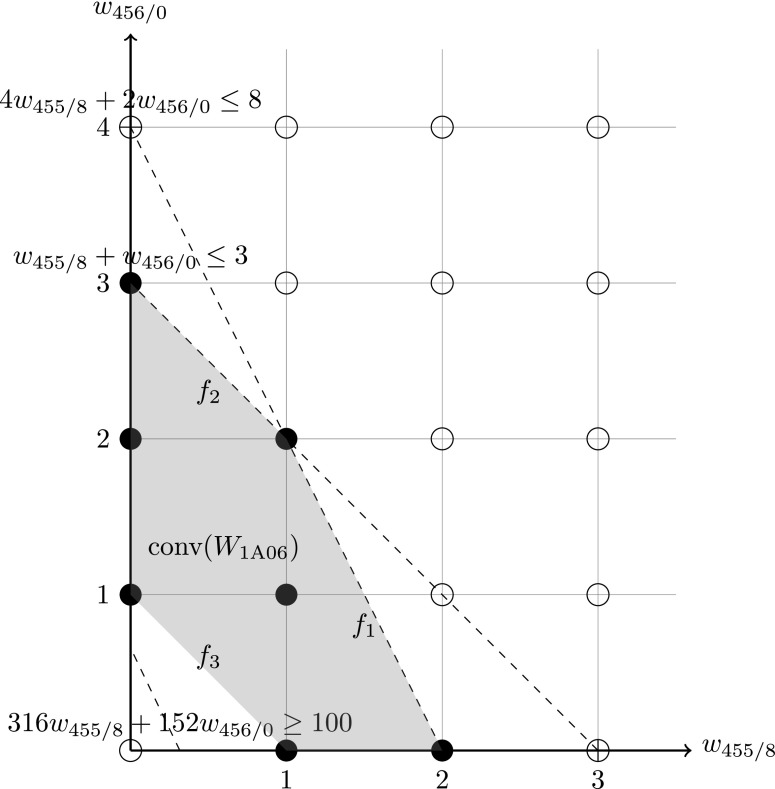
The local convex hull for train 1A06

Suppose for a train “1A06” with a passenger demand of 100 seats, train units of c455/8 (4-car, 316 seats) and c456/0 (2-car, 152 seats) are permitted. There are two restrictive factors for the coupling upper bounds, as shown in Table 2. If represented by explicit linear constraints, then two constraints are needed for ensuring the coupling upper bounds (before the variables are converted from *w* to *x*) as $4w_{455/8}+2w_{456/0} \le 8$ and $w_{455/8}+w_{456/0} \le 3$ and in this example they are not required to be disjunctive. Another constraint $316w_{455/8}+152w_{456/0} \ge 100$ is used for satisfying the passenger demand. On the other hand, we can enumerate all valid unit combinations as:

$$\begin{aligned} W_{\mathrm {1A06}}=\Big \{(w_{455/8},w_{456/0})\Big |(1,0),(2,0),(0,1),(1,1),(0,2),(1,2),(0,3)\Big \}, \end{aligned}$$

and compute its corresponding local convex hull:

$$\begin{aligned} {\mathrm {conv}}(W_{{\mathrm {1A06}}})=\left\{ w\in {\mathbb {R}}_+^2 \left| \begin{array}{l} f_1: 2w_{455/8}+w_{456/0}\le 4\\ f_2: w_{455/8}+w_{456/0}\le 3\\ f_3: w_{455/8}+w_{456/0}\ge 1 \end{array} \right. \right\} , \end{aligned}$$

which is a polytope with three nonzero facets $f_1,f_2,f_3$, giving three corresponding local convex hull constraints for train 1A06. Figure 2 gives an illustration on the above example. The filled points indicate valid unit combinations and the blank points are the invalid ones. The dashed lines give the constraints if explicit linear constraints are used and the shaded area is the convex hull of the valid combination points if the local convex hull is used. One can see that compared with the explicit demand satisfaction constraint ($316w_{455/8}+152w_{456/0} \ge 100$), the local convex hull has narrowed down the solution space shown at the bottom-left corner area in Fig. 2.

#### Previous studies on local convex hulls

Within the realm of railway rolling stock scheduling/assignment/circulation, the method of using explicitly computed local convex hulls to strengthen LP relaxation first appears in Schrijver [23] for a train unit scheduling problem, where since at most two commodities are involved, the convex hull computation is done in ${\mathbb {R}}_+^2$. This principle is also used in subsequent researches in train unit circulation in [2, 18]. Ziarati et al. [29] propose a similar method to generate cuts for a locomotive assignment problem. For $|K_j|=2$ they have established a relationship for their problem instance that the maximum number of nonzero facets of the lower envelope of ${\mathrm {conv}}(W_j)$ is 2*m* when $\max w_k^j=\frac{m^2+5m}{2}$ and $2m+1$ when $\max w^j_k=\frac{m^2+5m}{2}+1$, where $m \in {\mathbb {N}}_+$. They also show that this facet number will not exceed 4 when $|K_j|=3$ and $\max w_k^j\le 6$. Cacchiani et al. [6] give a local convex hull method for the train unit assignment problem to tighten the LP relaxation. Taking advantage of the problem’s feature that $u_j=2$, $v_k=1,\forall k \in K_j, \forall j \in {\mathcal {N}}$ and based on the combination sets in the form of (9), they find an explicit description of the dominants of the local convex hulls and apply this method to real-world instances where $|K_j|=10$. Also the characteristics of relevant integer polytopes have been studied [10]. In [14], local convex hulls are explicitly computed by standard QuickHull [3] to deal with combination-specific upper bounds and commodity compatibility, as well as to tighten the LP relaxation.

In all the instances above utilizing local convex hulls, it is either the case that the magnitudes of dimension $|K_j|$ and point number $|W_j|$ are small such that standard convex hull algorithms (e.g. QuickHull [3]) would suffice (e.g. [2, 14, 23]), or the problem has special features to allow an analytical description on the relevant dominants (e.g. [6]). However, the use of standard convex hull algorithms are not guaranteed for more difficult cases with higher dimensions and a larger number of points.

In this paper, first a further exploration on the feasibility of the local convex hull method subject to instances whose combination sets $W_j$ have higher dimensions (e.g. 5–20) and a larger number of points (e.g. hundreds to several thousands) will be given. Then a customized convex hull computation algorithm based on QuickHull will be presented. The computational feasibility of this method must have a limit when the number of points and space dimensions are getting large. Therefore empirical experiments will also be conducted to explore the feasibility range of the local convex hull method, mainly within the context of rolling stock scheduling.

## The structure of local convex hulls

In this section we assume that everything is based on the *n*-dimensional Euclidean space ${\mathbb {R}}^n$ (and its subset ${\mathbb {R}}_+^n$), associated with a commodity combination set $W \subset {\mathbb {Z}}_+^n$ containing |*W* | finite points representing all possible combinations from *n* available commodities indexed by $i=1,\ldots ,n$. The node name *j* will be omitted. Let *r* be the required demand or provision and let *u* be the shared resource upper bound for all combinations when *W* can also be represented by (9). A point in ${\mathbb {R}}^n$ is written as $w=(w_1,\ldots ,w_n)^T$. We are interested in explicitly computing ${\mathcal {H}}={\mathrm {conv}}(W)=\big \{w\in {\mathbb {R}}_+^n\big |H w \le h \big \}$, the convex hull of *W*.

Among all the combinations in *W*, consider those where only *one* commodity is used. In ${\mathbb {R}}_+^n$ the points representing them should lie precisely on the axes each associated with a single commodity and there will be only one nonzero entry in each of these points. A set containing all such points on axes is defined as

16 $$\begin{aligned} W^\prime =\big \{w\in W \big |w\text { is on an axis of }{\mathbb {R}}^n_+\big \}. \end{aligned}$$

Moreover, denote $W _i^\prime =\big \{w\in W^\prime \big |w\text { is on axis } i\big \},\forall i=1,\ldots ,n$, i.e. the set of points on axis *i*. We assume that for all commodities $i=1,\ldots ,n$, $W '_i\ne \emptyset$. Taking the TUSP for example, it is uncommon for a train to have an available unit type which can only be coupled with other types but is not allowed to run on its own. For a combination set *W* that can be represented by (9), this assumption means $\big \lceil \frac{r}{q_i} \big \rceil < \big \lfloor \frac{u}{v_i} \big \rfloor , \forall i=1,\ldots ,n$. The rare situation that this “single-commodity-presence” condition is not satisfied will be discussed in Sect. 2.3.

Now consider a combination set *W* that satisfies the above “single-commodity-presence” assumption. Within each $W'_i$, let

17 $$\begin{aligned} a_i= & {} \min _{w\in W^\prime _i} w_i,\quad \forall i=1,\ldots ,n, \end{aligned}$$

18 $$\begin{aligned} b_i= & {} \max _{w\in W^\prime _i} w_i,\quad \forall i=1,\ldots ,n. \end{aligned}$$

Then for each commodity (axis) $i=1,\ldots ,n$, $a_i$ and $b_i$ are the minimum and maximum flow amounts achieved by single commodity *i*. For *W* that can be represented by (9), $a_i=\big \lceil \frac{r}{q_i} \big \rceil$ and $b_i=\big \lfloor \frac{u}{v_i} \big \rfloor$, $\forall i=1,\ldots ,n$. The axis points having the entries of $a_i$ and $b_i$ are referred to as *end axis points*, denoted by

19 $$\begin{aligned} \underline{w}^i= & {} \big \{w\in W '_i\big |w_i=a_i\big \}=a_i e_i,\quad \forall i=1,\ldots ,n, \end{aligned}$$

20 $$\begin{aligned} \overline{w}^i= & {} \big \{w\in W '_i\big |w_i=b_i\big \}=b_i e_i,\quad \forall i=1,\ldots ,n, \end{aligned}$$

where $e_i\in {\mathbb {R}}^n$ is the unit vector with a 1 in the *i*-th entry and 0’s in the other entries. Let $V'=\{\underline{w}^1,\overline{w}^1,\ldots ,\underline{w}^n,\overline{w}^n\}$ be the set of all end axis points. Note that it is possible for a commodity *i* to have the case of $a_i=b_i$ such that $\underline{w}^i=\overline{w}^i$. Therefore $n\le |V'|\le 2n$.

**Fig. 3:**
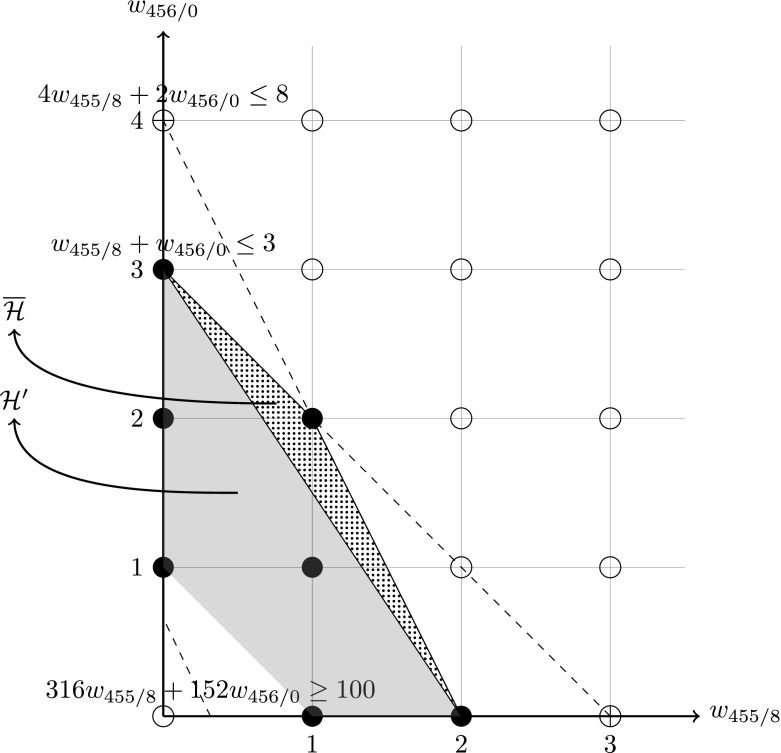
The main hull ${\mathcal {H}}'$, the up hull $\overline{{\mathcal {H}}}$ and the down hull (*empty*) for Train 1A06

We then define a polytope ${\mathcal {H}}'$ called the *main hull* as the convex hull of all end axis points, i.e. ${\mathcal {H}}'={\mathrm {conv}}(V')$. Since $V'\subseteq W$, then ${\mathcal {H}}'\subseteq {\mathcal {H}}$. Figure 3 shows an example of the main hull in the convex hull of the aforementioned example with Train 1A06. For $V'$ and ${\mathcal {H}}'$ we also have the following result.

### **Proposition 1**

$V'$ is the set of vertices of ${\mathcal {H}}'=conv (V')$.

### *Proof*

Let $V''$ be the set of vertices of ${\mathcal {H}}'$. First it is true that $V''\subseteq V'$[26]. Second any point in $V'$ cannot be expressed as a convex combination of any other points in $V'$, since they are the end axis points of the axes. Thus all points in $V'$ are vertices of ${\mathcal {H}}'$, or $V'\subseteq V''$. Therefore $V'=V''$. $\square$

The importance of ${\mathcal {H}}'$ lies in two aspects: First it can only have at most two nonzero facets; second it often contains a large proportion of the points in *W* for many problem instances in practice. The subsequent sections will elaborate the above two aspects.

### Nonzero facets of main hull ${\mathcal {H}}'$

#### Preliminaries

We will first show that ${\mathcal {H}}'$ has no more than two nonzero facets. For an optimization problem defined in ${\mathbb {R}}_+^n$, a zero facet represents a constraint in the form of $w_i\ge 0$ which is often satisfied implicitly. Here we briefly give some results in polyhedral combinatorics that will be used in deriving the above conclusion. For details of the results, see Nemhauser and Wolsey [20], Webster [26] and Mahjoub [17].

In ${\mathbb {R}}^n$, a set of *r* points $w^{(1)},\ldots ,w^{(r)}\in {\mathbb {R}}^{n\times 1}$ is affinely independent if the unique solution of the system of *r* variables $\sum _{i=1}^r \lambda _i w^{(i)}=\mathbf {0}$, $\sum _{i=1}^r\lambda _i=0$ is $\lambda _i=0, \forall i=1,\ldots ,r$. Equivalently, letting  be the matrix associated with the system, the points are affinely independent if ${\mathrm {rank}}\big (A_{\{w^{(1)},\ldots ,w^{(r)}\}}\big )=r$. *r* affinely independent points will uniquely define an $(r-1)$-flat (aka an $(r-1)$-dimensional affine set) which equals their affine hull. In particular, an $(n-1)$-flat is also called a hyperplane in ${\mathbb {R}}^n$. A set $\varPi$ in ${\mathbb {R}}^n$ is a hyperplane if and only if there exist scalars $\pi _0,\pi _1,\ldots ,\pi _n$, being not all zero, such that

21 $$\begin{aligned} \varPi =\left\{ w\in {\mathbb {R}}^n|\pi _1 w_1+\ldots +\pi _n w_n=\pi _0\right\} :=\left\{ w\in {\mathbb {R}}^n|\pi ^Tw=\pi _0\right\} . \end{aligned}$$

Each *r*-flat ($r=-1,0,\ldots ,n$) can be expressed as the intersection of $n-r$ hyperplanes, and so is the solution set of some system of $n-r$ linear equations.

Let $S^\prime$ be a subset of a finite set *S* in ${\mathbb {R}}^n$ such that ${\mathrm {aff}}(S^\prime ) \cap {\mathrm {conv}}(S \setminus S^\prime )=\emptyset$. Then ${\mathrm {conv}}(S^\prime )$ is a face of the polytope ${\mathrm {conv}}(S)$. If *F* is a nonempty $(r-1)$-face of ${\mathrm {conv}}(S)$, then there are *r* affinely independent points in $S\cap F$. A face *F* of ${\mathrm {conv}}(S)$ is a facet of ${\mathrm {conv}}(S)$ if ${\mathrm {dim}}(F)={\mathrm {dim}}({\mathrm {conv}}(S))-1$. Therefore a nonempty facet $F^*$ will imply ${\mathrm {dim}}({\mathrm {conv}}(S))$ affinely independent points in $S\cap F^*$.

A polyhedron $P\in {\mathbb {R}}^n$ is full-dimensional if ${\mathrm {dim}}(P)=n$. It has a unique minimal representation

22 $$\begin{aligned} P=\big \{w\in {\mathbb {R}}^n\big |(\pi ^i)^T w \le \pi _0^i, \forall i=1\ldots ,t\big \} \end{aligned}$$

as a finite set of *t* linear inequalities each representing a facet of *P*. For a polyhedron $P\in {\mathbb {R}}^n$ that is not full-dimensional with ${\mathrm {dim}}(P)=n-k,k>0$, its minimal representation is

23 $$\begin{aligned} P=\bigg \{w\in {\mathbb {R}}^n\bigg |\begin{array}{l} (\pi ^i)^T w = \pi _0^i, \forall i=1,\ldots ,k; \\ (\pi ^i)^T w \le \pi _0^i, \forall i=k+1,\ldots ,k+t. \end{array} \bigg \}, \end{aligned}$$

where each inequality of $i=k+1,\ldots ,k+t$ is from the equivalence class of inequalities representing a facet of *P*.

#### Number of nonzero facets of main hull

The following Theorem 1 gives the number of nonzero facets of ${\mathcal {H}}'={\mathrm {conv}}(V')$.

##### **Theorem 1**

When $a_i \ne b_i$ for at least one commodity *i*, then the main hull ${\mathcal {H}}'=conv (V')$ is full-dimensional and has precisely 2 nonzero facets represented by

24 $$\begin{aligned}&\frac{w_1}{a_1}+\cdots +\frac{w_n}{a_n}\ge 1, \end{aligned}$$

25 $$\begin{aligned}&\frac{w_1}{b_1}+\cdots +\frac{w_n}{b_n}\le 1, \end{aligned}$$

and has at most *n* zero facets represented by

26 $$\begin{aligned} w_i\ge 0,\quad \forall i=1,\ldots ,n. \end{aligned}$$

When $a_i=b_i,\forall i=1,\ldots ,n$, then ${\mathcal {H}}'$ degenerates into an $(n-1)$-face per se represented by a single nonzero hyperplane

27 $$\begin{aligned} \frac{w_1}{a_1}+\cdots +\frac{w_n}{a_n}=1, \end{aligned}$$

and *n* zero facets each corresponding to one of the inequalities as in (26).

##### *Proof*

To get the very details of ${\mathcal {H}}$, we use a straightforward way in proving based on the points in *W*.

1. First consider the case $a_i \ne b_i$ for at least one commodity *i*.

Let $I=\{1,\ldots ,n\}$. Divide the commodities into two groups as $I^{\ne }=\{i\in I|a_i\ne b_i\}$ and $I^==\{i\in I|a_i=b_i\}$ such that $I=I^{\ne }\cup I^=$, $1\le |I^{\ne }|\le n$ and $0\le |I^=|\le n-1$. Since $|I^{\ne }|\ge 1$, without loss of generality, $\exists k\in I^{\ne }$ such that we can find $n+1$ points from ${\mathcal {H}}'$ as $\underline{w}^1,\ldots ,\underline{w}^k,\overline{w}^k,\ldots ,\underline{w}^n$. Then we have28which shows the $n+1$ points are affinely independent. Therefore ${\mathcal {H}}'$ is full-dimensional with ${\mathrm {dim}}({\mathcal {H}}')=n$. Its minimal representation is a finite set of inequalities each corresponding to a facet of ${\mathcal {H}}'$.

Based on the $n<|V'|\le 2n$ points in $V'$, we can find all possible facets of ${\mathrm {conv}}(V')$ by enumerating the ${|V'| \atopwithdelims ()n}$ combinations and checking their validity. Two cases are identified and will be discussed separately.

Case 1: Collect the *n* points by taking one and only one from each of the *n* axis as $w^{(1)},\ldots ,w^{(n)}$ such that $w^{(i)}\in \{\underline{w}^i,\overline{w}^i\},\forall i\in I$. The *n* points are affinely independent since it can be verified that ${\mathrm {rank}}\big (A_{\{w^{(1)},\ldots ,w^{(n)}\}}\big )=n$. Suppose the hyperplane formed by them is $\{w\in {\mathbb {R}}^n|\pi ^Tw=\pi _0\}, (\pi _0,\pi )\ne \mathbf {0}$. Then the solution of the system $\pi ^Tw^{(i)}=\pi _0,\forall i\in I$ is $(\pi _0,\pi )=c\Big (1,\frac{1}{w_1^{(1)}},\ldots ,\frac{1}{w_n^{(n)}}\Big )^T,\forall c\in {\mathbb {R}}\setminus \{0\}$. Letting $c=1$ gives a convenient expression of this nonzero hyperplane:

29 $$\begin{aligned} \frac{w_1}{w_1^{(1)}}+\cdots +\frac{w_n}{w_n^{(n)}}=1. \end{aligned}$$

If $w^{(i)}=\underline{w}^i,\forall i\in I$, then (29) becomes $\frac{w_1}{a_1}+\cdots +\frac{w_n}{a_n}=1$, or in short as $(a^{-1})^Tw=1$, which supports ${\mathrm {conv}}(V')$ since (i) $(a^{-1})^Tw\ge 1, \forall w\in V'$; (ii) There are *n* affinely independent points $\underline{w}^1,\ldots ,\underline{w}^n \in V'$ such that $(a^{-1})^T\underline{w}^i=1,\forall i\in I$. Therefore, $(a^{-1})^Tw\ge 1,w\in {\mathbb {R}}^n$ defines a nonzero facet of ${\mathrm {conv}}(V')$ as given by (24). By similar reasoning, it can be concluded that if $w^{(i)}=\overline{w}^i,\forall i\in I$, then (29) will give another nonzero facet of ${\mathrm {conv}}(V')$ as given by (25).

If neither $w^{(i)}=\underline{w}^i$, $\forall i\in I$ nor $w^{(i)}=\overline{w}^i$, $\forall i\in I$ (which can only happen when $|I^{\ne }|\ge 2$, since when $|I^{\ne }|=1$, the only two possible combinations are still $w^{(i)}=\underline{w}^i$ and $w^{(i)}=\overline{w}^i$, $\forall i\in I$), the hyperplane given by (29) cannot yield any valid inequality. To see this, suppose $|I^{\ne }|\ge 2$ and not all commodities in $I^{\ne }$ are from the same group of $\{\underline{w}^i\}_{\forall i\in I^{\ne }}$ or $\{\overline{w}^i\}_{\forall i\in I^{\ne }}$. Divide the commodities in $I^{\ne }$ into two nonempty subsets as $I^{\ne }=\underline{I}^{\ne }\cup \overline{I}^{\ne }$ such that $i\in \underline{I}^{\ne }$ if $w^{(i)}=\underline{w}^i$ and $i\in \overline{I}^{\ne }$ if $w^{(i)}=\overline{w}^i$. Then the hyperplane given by (29) would be

30 $$\begin{aligned} h(w)=\sum _{i\in \underline{I}^{\ne }}\frac{w_i}{a_i}+\sum _{i\in \overline{I}^{\ne }}\frac{w_i}{b_i}+\sum _{i\in I^=}\frac{w_i}{a_i}-1=0. \end{aligned}$$

Now we can always find at least two points in $V'$ as $\underline{w}^p=a_p e_p, p\in \overline{I}^{\ne }$ and $\overline{w}^q=b_q e_q, q\in \underline{I}^{\ne }$, such that $h(\underline{w}^p)=\frac{a_p}{b_p}-1<0$ and $h(\overline{w}^q)=\frac{b_q}{a_q}-1>0$. Therefore (30) cannot yield any valid inequality and is not facet-defining for ${\mathrm {conv}}(V')$.

Case 2: Collect the *n* end axis points such that the points from *k* of the axes will not be present. Note that since each axis only has at most two distinct points and there are *n* axes, then points from an axis $p\in I$ are absent if and only if another axis $q\in I^{\ne }$ has both $\underline{w}^q$ and $\overline{w}^q$ collected. Thus for a given set of collected points it is true that $1\le k \le |I^{\ne }|$, i.e. there will be no more than $|I^{\ne }|$ absent axes or “double-collected” axes.

Suppose $p_1,\ldots ,p_k\in I$ are absent and $q_1,\ldots ,q_k\in I^{\ne }$ are correspondingly “double-collected” and let the *n* points be $w^{(1)},\ldots ,w^{(n)}$ such that $w^{(i)}\in \{\underline{w}^i,\overline{w}^i\}, \forall i\in I\setminus \{p_1,\ldots ,p_k\}$, $w^{(p_j)}\in \{\underline{w}^{q_j},\overline{w}^{q_j}\}$ and $w^{(p_j)}\ne w^{(q_j)}$, $\forall j=1,\ldots ,k$. Then by a similar reasoning as in (28) and noticing there are *k* rows of all zeros in $A_{\{w^{(1)},\ldots ,w^{(n)}\}}$ corresponding to the *k* missing axes, we have ${\mathrm {rank}}\big (A_{\{w^{(1)},\ldots ,w^{(n)}\}}\big ) = n-k+1$ .

When $k=1$, the above rank is *n* showing the *n* points are still affinely independent. Suppose they form a hyperplane $\{w\in {\mathbb {R}}^n|\pi ^Tw=\pi _0\}, (\pi _0,\pi )\ne \mathbf {0}$. Then the solution of the system $\pi ^Tw^{(i)}=\pi _0,\forall i\in I$ is $\pi _{p_1}=c,\forall c\in {\mathbb {R}}\setminus \{0\},\pi _i=0,\forall i\ne p_1$, which however leads to a zero facet represented by $w_{p_1}\ge 0$.

When $1<k\le |I^{\ne }|$, the above rank is less than *n*, showing the *n* points are no longer affinely independent. They can be disregarded in the search for the facets of ${\mathcal {H}}'$. We will show that none of the facets can be derived from them. Suppose ${\mathrm {aff}}\{w^{(i)}\}_{i=1}^n \cap {\mathrm {conv}}(V' \setminus \{w^{(i)}\}_{i=1}^n)=\emptyset$ such that ${\mathrm {conv}}\{w^{(i)}\}_{i=1}^n$ defines a face *F* of ${\mathcal {H}}'$, then we have ${\mathrm {dim}}(F) = {\mathrm {dim}}({\mathrm {conv}}\{w^{(i)}\}_{i=1}^n) = {\mathrm {dim}}({\mathrm {aff}}({\mathrm {conv}}\{w^{(i)}\}_{i=1}^n)) = {\mathrm {dim}}({\mathrm {aff}}\{w^{(i)}\}_{i=1}^n) < n-1$. In fact here the points can only yield some zero faces of dimensions less than $n-1$ as the intersections of some zero facets.

Now we can have an exact description of the zero facets in ${\mathcal {H}}'$. Since the absence of points from one and only one axis *p* leads to a zero facet $w_p\ge 0$, the zero facets are solely determined by the axes that are absent in all possible point combinations with $k=1$. We only focus on the cases when $n>1$ as the condition $n=1$ is trivial. If $|I^{\ne }|>1$, when an axis $p\in I$ is absent there is always at least a $q\in I^{\ne }$ available to be “double-collected”, including those $p\in I^{\ne }$ with a $q\in I^{\ne }\setminus \{p\}$. So there are *n* zero facets $w_i\ge 0,i\in I$. If $|I^{\ne }|=1$, however, there is no axis to be “double-collected” if the only axis $p\in I^{\ne }$ is absent. Thus there are $n-1$ zero facets $w_i\ge 0,\forall i\in I^=$.

(2) Second consider the case $a_i=b_i$, $\forall i=1,\ldots ,n$, such that $|V'|=n$ and $|I^{\ne }|=0$.

Let the vertex set $V'=\{w^{(1)},\ldots ,w^{(n)}\}$, which contains *n* affinely independent points. We have ${\mathrm {dim}}({\mathcal {H}}') = {\mathrm {dim}}({\mathrm {conv}}\{w^{(i)}\}_{i=1}^n) = {\mathrm {dim}}({\mathrm {aff}}({\mathrm {conv}}\{w^{(i)}\}_{i=1}^n)) = {\mathrm {dim}}({\mathrm {aff}}\{w^{(i)}\}_{i=1}^n) = n-1.$ Therefore ${\mathcal {H}}'$ is not a full-dimensional polytope. Since ${\mathrm {dim}}({\mathcal {H}}')=n-1$, its minimal representation consists of (i) a finite set of inequalities each corresponds to a facet of ${\mathcal {H}}'$ and (ii) an equality that is attained by all points in ${\mathcal {H}}'$.

The equality is just the hyperplane $\varPi _0={\mathrm {aff}}\{w^{(1)},\ldots ,w^{(n)}\}=\{w \in {\mathbb {R}}^n | \sum _{k=1}^n \frac{w_k}{a_k} =1 \}$ as given by (29).

Facets of ${\mathcal {H}}'$ are $(n-2)$-faces each being a convex hull formed by $n-1$ affinely independent points in $V'$. Moreover, each facet is also associated with a supporting $(n-2)$-flat which is the affine hull of the same $n-1$ points that forms the facet. The number of facets will not exceed *n* as at most *n* such point combinations can be from $V'$ by each time removing a point in axis *p*, $\forall p\in I$. Now consider the *n* flats of dimension $n-2$: $\varPhi _p={\mathrm {aff}}\{w^{(i)}\}_{\forall i\in I\setminus \{p\}}, \forall p \in I$ as intersections of two non-parallel hyperplanes $\varPi _0$ and $\varPi _p$

31 $$\begin{aligned} \varPhi _p:\ \left\{ \begin{array}{l} \varPi _0:\frac{w_1}{a_1}+\cdots +\frac{w_n}{a_n}=1, \\ \varPi _p:w_p=0. \end{array}\right. \ \forall p \in I. \end{aligned}$$

Let $h_0(w)=\frac{w_1}{a_1}+\cdots +\frac{w_n}{a_n}-1$ and $h_p(w)=w_p$, $\forall p \in I$. Then $\varPhi _p$ supports ${\mathcal {H}}'$, $\forall p \in I$ since (i) $h_0(w^{(i)})=0$, $h_p(w^{(i)})\ge 0$, $\forall i\in I$ and (ii) there are $n-1$ affinely independent points $\{\underline{w}^j\}_{j\in I\setminus \{p\}}\in {\mathcal {H}}'$ such that $h_0(\underline{w}^{j})=0$ and $h_p(\underline{w}^j)=0$. Therefore, apart from the redundant valid inequality from $\varPi _0$, the remaining valid inequalities representing facets of ${\mathcal {H}}'$ are just $w_p\ge 0,\forall p\in I$, as given by (26). $\square$

Now we have the exact description of the main hull, as (it is also valid for degenerated ${\mathcal {H}}'$)

32 $$\begin{aligned} {\mathcal {H}}'=\Bigg \{w \in {\mathbb {R}}_+^n \Bigg | \frac{w_1}{a_1}+\cdots +\frac{w_n}{a_n}\ge 1, \frac{w_1}{b_1}+\cdots +\frac{w_n}{b_n}\le 1 \Bigg \}. \end{aligned}$$

It can be verified that this is actually a frustum of a simplex formed by the intersection of an *n*-simplex $\Big \{w \in {\mathbb {R}}_+^n \Big | (b^{-1})^T w \le 1 \Big \}$ and a halfspace $\Big \{w \in {\mathbb {R}}^n \Big | (a^{-1})^T w \ge 1 \Big \}$. See [25] for details on a frustum of a simplex.

Similar conclusions can be made by analogy for other integer multicommodity flow problems suitable for the local convex hull method. For example, for the Multivehicle Tanker Scheduling Problem, the corresponding main hull is then an *n*-simplex $\Big \{w \in {\mathbb {R}}_+^n \Big | (b^{-1})^T w \le 1 \Big \}$ with *n* zero facets and only one nonzero facet.

### Number of outside points

Now consider the points in *W* outside the main hull ${\mathcal {H}}'$ which we refer to as *outside points* as the remaining points in $W \setminus W'$. Since ${\mathcal {H}}'$ has only at most two nonzero facets, for an outside point $w \in W \setminus W'$, it is either in $\overline{W}=\big \{w \in W\big |(b^{-1})^T w > 1\big \}$ or in $\underline{W}=\big \{w \in W \big | (a^{-1})^T w < 1\big \}$. Thus we define an *up hull* and a *down hull* as

33 $$\begin{aligned} \overline{{\mathcal {H}}}= & {} \bigg \{w \in {\mathcal {H}}\bigg |\frac{w_1}{b_1}+\cdots +\frac{w_n}{b_n}>1\bigg \}, \end{aligned}$$

34 $$\begin{aligned} \underline{{\mathcal {H}}}= & {} \bigg \{w \in {\mathcal {H}}\bigg |\frac{w_1}{a_1}+\cdots +\frac{w_n}{a_n}<1\bigg \}. \end{aligned}$$

such that $W=W' \cup \overline{W}\cup \underline{W}$ and ${\mathcal {H}}={\mathcal {H}}' \cup \overline{{\mathcal {H}}}\cup \underline{{\mathcal {H}}}$. Note that $\overline{{\mathcal {H}}}$ and $\underline{{\mathcal {H}}}$ are convex sets each formed by a polytope without one of its facet. It is also not difficult to verify that $\overline{W}=\overline{{\mathcal {H}}}\cap {\mathbb {Z}}_+^n$ and $\underline{W}=\underline{{\mathcal {H}}}\cap {\mathbb {Z}}_+^n$. Figure 3 gives an example of the upper hull and the down hull (empty) in the convex hull from Sect. 1.2.2.

If the number of outside points is of a moderate size, and the dimension *n* is appropriately small, then the entire convex hull ${\mathcal {H}}$ can be computed based on ${\mathcal {H}}'$, $\overline{W}$ and $\underline{W}$ by some convex hull algorithms. This might be more efficient and less intractable than computing ${\mathcal {H}}$ directly starting with the given points in *W*. In this part we will briefly explore the number of outside points $\overline{W}$ and $\underline{W}$ and leave the discussion of this convex hull algorithm to Sect. 3.

#### Two special conditions

The number of outside points can be analytically determined under two special conditions, i.e. when all commodities are incompatible and when *u* (or *r*) as given in (9) is a multiple of all elements in *v* (or *q*).

For an instance with commodity compatibility relations as mentioned in Sect. 1.1, the combination point enumeration and outside point counting can be decomposed into subsets of compatible commodities. Let $I_1,\ldots ,I_S\subset I=\{1,\ldots ,n\}$ be the subsets of commodities each containing compatible commodities such that $I_{s_1} \cap I_{s_2} = \emptyset , \forall s_1 \ne s_2$. With respect to each subset $s=1,\ldots ,S$, we have the combination set $W_s$ such that $\bigcup _{s=1}^S W_s=W$, and the outside points $\overline{W}_s,\underline{W}_s$ defined by $\sum _{i \in I_s}\frac{w_i}{b_i}>1$ or $\sum _{i \in I_s}\frac{w_i}{a_i}<1$ such that $\bigcup _{s=1}^S \overline{W}_s=\overline{W}$ and $\bigcup _{s=1}^S \underline{W}_s=\underline{W}$. In fact in this case ${\mathcal {H}}={\mathrm {conv}}(W)$ can be constructed by “wrapping the projections” of the sub-hulls of each subset. Moreover, when commodities are all incompatible with each other (which can be found in real-world instances in train unit scheduling), the following Proposition 2 states that the convex hull can be given directly by the main hull since $\underline{W}$ and $\overline{W}$ are both empty.

##### **Proposition 2**

For a combination set *W* where all commodities $k \in K$ are incompatible with each other, *W* will only have axis points such that $W=W'$ and ${\mathcal {H}}={\mathcal {H}}'$.

In addition, consider a combination set $W=\big \{w\in {\mathbb {Z}}_+^n\big |q^T w \ge r, v^Tw \le u\big \}$ that can be defined by (9). Two simplices can be found such that their difference contains the up hull as $\overline{{\mathcal {H}}}\subseteq \overline{P}= S_{vu} \setminus S_b$, where $S_{vu}=\big \{w\in {\mathbb {R}}^n_+\big |v^Tw\le u\big \}, S_b=\big \{w\in {\mathbb {R}}^n_+\big |(b^{-1})^Tw\le 1\big \}$ and $b_i=\big \lfloor \frac{u}{v_i}\big \rfloor ,\forall i=1,\ldots ,n$. The situation is slightly more complicated for the down hull where $\underline{{\mathcal {H}}}\subseteq \underline{P}=S_a \setminus S_{qr} \cup \varPi _{qr} \setminus \varPi _a$, where $S_{qr}=\big \{w\in {\mathbb {R}}_+^n\big |q^Tw\le r\big \}$, $S_a=\big \{w\in {\mathbb {R}}_+^n\big |(a^{-1})^Tw\le 1\big \}$, $\varPi _{qr}=\big \{w\in {\mathbb {R}}_+^n\big |q^Tw = r\big \}$, $\varPi _a=\big \{w\in {\mathbb {R}}_+^n\big |(a^{-1})^Tw=1\big \}$, and $a_i=\big \lceil \frac{r}{q_i} \big \rceil ,\forall i=1,\ldots ,n$.

Here we have the following properties for the emptiness of $\overline{P}$ and $\underline{P}$, due to the fact that $a_i=\big \lceil \frac{r}{q_i} \big \rceil , b_i=\big \lfloor \frac{u}{v_i}\big \rfloor ,\forall i=1,\ldots ,n$.

##### **Proposition 3**

For a combination set *W* that can be defined by (9), if *u* is a multiple of all $v_i,i=1,\ldots ,n$, then $\overline{P}=S_{vu} \setminus S_b =\emptyset$ such that $\overline{{\mathcal {H}}}=\emptyset$; if *r* is a multiple of all $q_i,i=1,\ldots ,n$, then $\underline{P}=S_a \setminus S_{qr} \cup \varPi _{qr} \setminus \varPi _a=\emptyset$ such that $\underline{{\mathcal {H}}}=\emptyset$.

The above condition can often happen in real-world instances. Taking the train unit scheduling problem for example, where the upper bound *u* is measured in number of cars and $v_i$ are the number of cars of unit type *i*, there can be many trains with *u* as the multiple of all types’ car numbers in the instances both from Southern Railways and ScotRail. If *u* is measured in number of units, as in [6], then this condition for the upper hull will always hold. Also note that when $\overline{P}\ne \emptyset$ it is still possible that $\overline{{\mathcal {H}}}=\emptyset$. On the other hand, *r*, as the demand measured in passenger numbers, can hardly be a multiple of all $q_i$, which are the numbers of seats of unit types $i=1,\ldots ,n$. Although $\underline{P}$ can hardly be empty, the size of $|\underline{W}|$ tends to be very small, even often be zero, since a non-empty $\underline{P}$ does not necessarily imply a non-empty $\underline{W}$. Figure 3 is a good example. This fact can also be observed in the experiments to be reported in Sect. 4.

#### Empirical experiments

Pragmatically for a given set *W* with points *w*, it is sufficient to determine the number of outside points simply by checking the values of $h_a(w)=(a^{-1})^T w - 1$ and $h_b(w)=(b^{-1})^T w - 1$. If $h_a(w)<0$ then $w \in \underline{W}$ and if $h_b(w)>0$ then $w\in \overline{W}$. A series of computational experiments were conducted to show the characteristics on the number of outside points under different circumstances. They will be reported in Sect. 4 in detail.

**Fig. 4:**
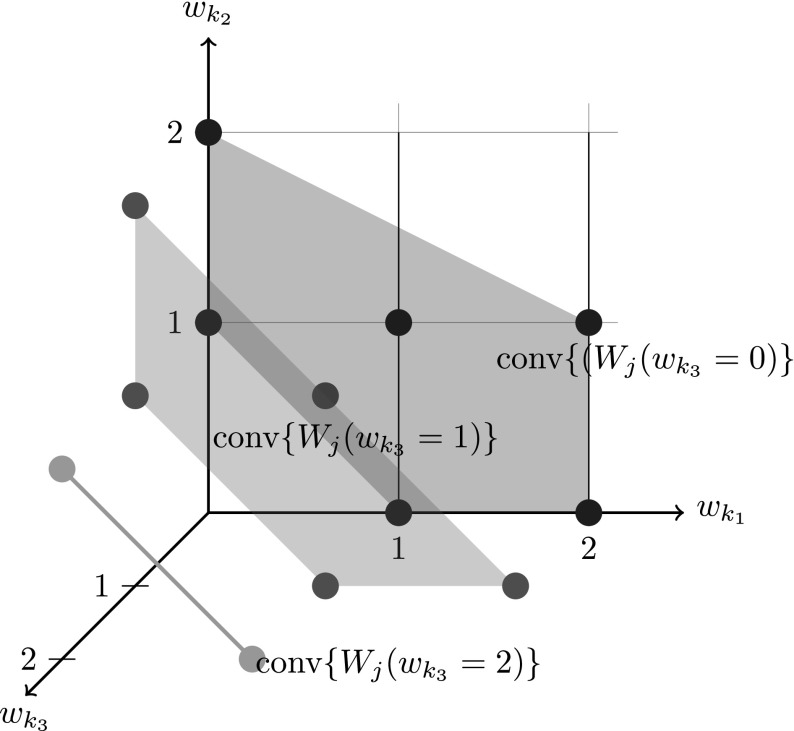
Example of non-standalone type $k_3$ with three branched convex hulls in *blue* ($w_{k_3}=0$), *red* ($w_{k_3}=1$) and *green* ($w_{k_3}=2$) (Color figure online)

### Non-standalone types

Although very rare under train unit scheduling scenarios, theoretically we cannot eliminate the possibility that a unit type can only be used with other types, e.g. as a result of a demand/upper bound pair at certain levels. We call this unit type that cannot satisfy the “single-commodity-presence” assumption for a train as a “non-standalone” type. When a non-standalone type exists, it is not possible to straightforwardly construct the main hull and the customized convex hull algorithm to be introduced in Sect. 3 will thus be inapplicable. However, it is still possible to compute ${\mathcal {H}}$ using the standard convex hull algorithm as given in [3].

Nonetheless, if there is only one non-standalone type, it is possible to modify the original problem to keep “single-commodity-presence” condition. Let $k^*$ be the unique non-standalone type at train *j*. The basic idea is to branch the problem into subproblems by fixing the possible values of $w_{k^*}$ used at *j*. For each fixed $w_{k^*}$, the convex hulls on other types can be constructed in a subspace one dimension less due to the absence of $k^*$, where the “single-commodity-presence” condition will be preserved. Thus the original problem will be branched into subproblems. When there is more than one train having unique non-standalone types, a tree structure is required to organize the subproblems as computationally independent nodes on the tree. The tree can be constructed in advance given the input information (where all convex hull constraints will be given once-for-all) or embedded into the branch-and-bound tree (where the convex hull constraints on trains with standalone types will have to be computed “on-line” during branching).

We use an example to illustrate the above method. Suppose at train *j* we have the unit combination set

$$\begin{aligned} W_j= & {} \big \{(1,0,0),(2,0,0),(0,1,0),(1,1,0),(2,1,0),(0,2,0)\ \\&(1,0,1),(2,0,1),(0,1,1),(1,1,1),(0,2,1),\\&(1,0,2),(0,1,2) \big \} \end{aligned}$$

that does not meet the single-commodity-presence condition due to the non-standalone type $k_3$. Then the original problem can be split into three subproblems with $w_{k_3}=0,1,2$, or in the model, with constraints

35 $$\begin{aligned} \sum _{p \in P_j^{k_3}} x_p=0,1,2 \end{aligned}$$

added respectively. For each fixed value on $w_{k_3}$, the points in subspaces over ${\mathbb {R}}_+^{k_1 \times k_2}$ satisfy the single-commodity-presence condition. Their local convex hulls to be used in the subproblem models are:

$$\begin{aligned} {\mathrm {conv}}\{W_j(w_{k_3}=0)\}= & {} \left\{ w\in {\mathbb {R}}_+^2 |w_{k_1} + w_{k_2} \ge 1, w_{k_1} + 2w_{k_2} \le 4, w_{k_1} \le 2 \right\} ,\\ {\mathrm {conv}}\{W_j(w_{k_3}=1)\}= & {} \left\{ w\in {\mathbb {R}}_+^2 | w_{k_1} + w_{k_2} \ge 1,w_{k_1} + w_{k_2} \le 2\right\} , \text { and } \\ {\mathrm {conv}}\{W_j(w_{k_3}=2)\}= & {} \left\{ w\in {\mathbb {R}}_+^2 | w_{k_1}+w_{k_2}=1 \right\} . \end{aligned}$$

Figure 4 gives an illustration on the three convex hulls from the above example.

If there are multiple non-standalone types at a train, theoretically it is still possible to enumerate all possibilities over the non-standalone types and for each case a convex hull in the subspace over the other types can be constructed satisfying the single-commodity-presence condition. However it is unclear about its practicality under this more difficult circumstance as the scheme may become quite complex to implement and the resulting tree may be too huge to tackle with. We will leave the relevant investigation to future work.^1^

## A customized QuickHull algorithm to compute local convex hulls

In this section we will describe how to use a customized QuickHull algorithm adapted from [3] to exactly compute the convex hull ${\mathcal {H}}={\mathrm {conv}}(W)$ based on the main hull ${\mathcal {H}}'$ and the outside points in $\underline{W},\overline{W}$. QuickHull is an algorithm that is theoretically able to compute the convex hull of a finite set of points in ${\mathbb {R}}^n$. The computational performance of the QuickHull algorithm is usually problem-specific, although it is reported that a generic QuickHull is suitable for medium/large-sized inputs for $n\le 8$ while not suitable for medium-sized inputs for $n\ge 9$ [24]. Its rationale is based on the following simplified Grünbaum’s Beneath-Beyond Theorem [3, 13].

### **Theorem 2**

(Grünbaum) Let ${\mathcal {H}}$ be a convex hull in ${\mathbb {R}}^n$, and let *w* be a point in ${\mathbb {R}}^n \setminus {\mathcal {H}}$. Then *F* is a facet of ${\mathrm {conv}}\big (w \cup {\mathcal {H}}\big )$ if and only if

(i) F is a facet of ${\mathcal {H}}$, and w is below F; or
(ii) F is not a facet of ${\mathcal {H}}$, and its vertices are w and the vertices of a ridge (i.e. an $(n-2)$-face) of ${\mathcal {H}}$ with one incident facet below w and the other incident facet above w.

A point’s position as being above or below a hyperplane/facet is defined by giving the hyperplane/facet an orientation as their outer normal’s direction and if the signed distance of a point to the hyperplane/facet is positive (negative), then the point is said to be above (below) the hyperplane/facet. A facet is said to be visible to a point if the point is above it. A realizable (feasible) point is below every hyperplane/facet.

For a given set of points, QuickHull first selects a non-degenerated subset of them to form an initial simplex as their convex hull. If possible, this initial simplex will be selected such that it will cover as many points as possible by choosing the points with either a maximum or minimum coordinate. Each point outside the initial simplex will be assigned to one and only one of its visible facet(s) of this simplex. Then the following recursive processes will be applied to each facet of the updated hull with its associated outside points. Within each facet, one of its associated point (generally the “furthest” one) will be selected. New facets will be made by joining this point and all horizontal ridges that enclose all visible facets of this point. A process called partitioning will either reallocate each outside points associated with one visible facet to a new facet or include that point into the hull. Then the point’s visible facets will be discarded. This will be repeated for the hull with updated facets until all facets have empty outside point sets. See [3] for details of the QuickHull algorithm.

From the view of the QuickHull algorithm, it can be seen that for the case of commodity combination set *W*, the “up” and “down” points in $\overline{W}$ and $\underline{W}$ should be above the hyperplanes $(b^{-1})^Tw=1$ and $(a^{-1})^Tw=1$ respectively. Moreover, the main hull ${\mathcal {H}}'$, as a frustum of a simplex, should be an ideal alternative for the initial simplex in the QuickHull’s first stage (by Theorem 2, any full-dimensional convex polytope inside ${\mathcal {H}}$ would do the job). Moreover, when ${\mathcal {H}}'$ is chosen as the “initial simplex”, there are initially only two visible facets, the two nonzero facets with respect to $(b^{-1})^Tw=1$ and $(a^{-1})^Tw=1$, to be further processed possibly in parallel with the “up” and “down” points. Therefore, given the “initial simplex” as the analytically known main hull ${\mathcal {H}}'$, it is only needed to apply standard QuickHull to $\underline{W}$ and $\overline{W}$ in parallel to compute ${\mathcal {H}}$, which might be more efficient than applying it to *W* directly if $W'$ contains most of the points in *W*. Based on the above principle, a customized “2-facet” QuickHull algorithm is given in Algorithm 1, which can be regarded as a tailored version of [3] only differing in how to construct the “initial simplex”.
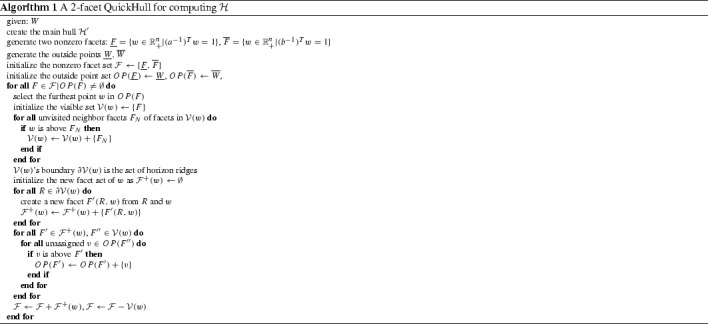


In practice, implementing Algorithm 1 from scratch may require a considerable amount of work. Pragmatically having the free and highly efficient QuickHull program available from its official website [24], one may consider the following alternative Algorithm 2 by using the official QuickHull program from [24]. Compared with Algorithm 1 which computes the local convex hulls all by itself, Algorithm 2 assumes that a tool for computing convex hulls is at hand (e.g. standard QuickHull) and it computes the local convex hulls indirectly using this tool.
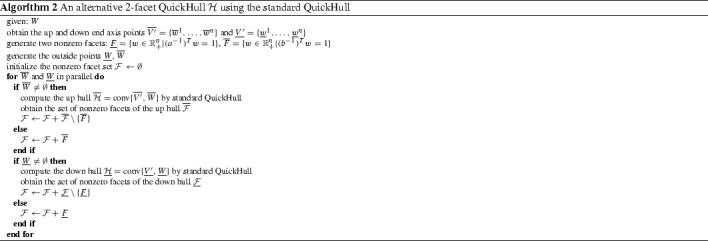


*A special case in computing local convex hulls for instances with incompatible commodities* QuickHull is very sensitive to increasing dimensions. When a node is to be served by commodities divided into subsets $I_1,\ldots ,I_S \in I=\{1,\ldots ,n\}$ of compatible ones, it is possible to first compute the convex hulls ${\mathcal {H}}_s$ (either by the customized Algorithm 1 or by a standard QuickHull) within each subset *s* in spaces with lower dimensions ${\mathbb {R}}_+^{|I_s|}$, $\forall s=1,\ldots ,S$ and merge the above “sub-hulls” into the target one as ${\mathcal {H}}=\bigcup _{s=1}^S {\mathcal {H}}_s$. One way of performing such merging is to construct ${\mathcal {H}}$ based on the vertices of all sub-hulls in ${\mathbb {R}}^n$. There is a nice property such that vertices from different sub-hulls are orthogonal. It is not apparent if this method can be competitive with Algorithm 1 subject to different *n*. We will leave this alternative method to future research.

## Computational experiments on local convex hulls

### Feasibility range of local convex hull method

The feasibility range of the local convex hull method is an interesting topic, which asks to what limit in terms of the input parameters it will become impractical or intractable to compute ${\mathcal {H}}$ explicitly based on points in *W* (standard QuickHull) or indirectly based on ${\mathcal {H}}'$, $\overline{W}$ and $\underline{W}$ (2-facet QuickHull). This would be very useful to guide the modeling and solving for some upgraded or new problem instances. For example, in [14] the local convex hulls are easily computed by QuickHull since $|K_j| \le 4, |W_j| \le 9$, $\forall j \in {\mathcal {N}}$. However, if a new instance requires that $|K_j|=10$ for train *j* as a result of the electrification upgrades in some routes such that electric units can also run on the routes that were previously only covered by diesel units, then it is important to see if the original methods are still practical.

However the problem of determining the feasibility of convex hull algorithms is complicated itself. Generally the difficulty of convex hull computation grows drastically when the dimension *n* becomes large, as a result of the complex components and structural sophistication of high dimensional polytopes. The Upper Bound Theorem [19] states that the total complexity of the convex hull of *m* points in *n* dimensions is $O(m^{\lfloor n/2 \rfloor })$. Particularly for the QuickHull algorithm, the authors in [3] conjecture that the time complexity of QuickHull for *m* points (with *p* processed points) in dimension *n* is in average $O(m \log m)$ when $n \le 3$ and $O(m f_p / p)$ when $n \ge 4$, where $f_p$ is the maximum number of facets for *p* vertices. According to [19], in the worse case the complexity of $f_p$ can be $O(p^{\lfloor n/2 \rfloor })$. Therefore the time complexity of QuickHull in computing *m* points with *p* processed points can be $O(mp^{\lfloor n/2 \rfloor -1})$ for $n \ge 4$. This gives a theoretical way in estimating the time complexity of QuickHull subject to different inputs of *m*, *n* and *p*.

In practice, if the number of outside points are within a reasonable range, like in many practical problems in rolling stock scheduling, the computation may still be possible for some not too large *n*. Within possibly the most difficult artificial conditions (with space dimensions no greater than 11) in the context of train unit scheduling, a series of computational experiments have been conducted giving some positive results on the feasibility range of the local convex hull method. Since there is evidence that those problems are not challenging enough for standard QuickHull, the convex hull computation results on the above experiments will not be reported for every individual instance but only for the possibly hardest ones among them. For the same reason, we only report the number of points rather than the total complexity $O(mp^{\lfloor n/2 \rfloor }$) or computational times for those experiments. Investigations on to what extent local convex hulls are still computable for a broader range of problem instances arising in various integer multicommodity flow problems cannot be comprehensively covered in this paper. Nevertheless, to check the performance of the 2-facet QuickHull under more difficult circumstances, we will report a series of experiments on more challenging artificial instances based on the fleets of ScotRail and Southern Railway by increasing the space dimensions from 2 to 21 and using both the standard and the “2-facet” QuickHull methods. Various results such as the numbers of points, computational times and complexity will be reported in detail.

### Number of points in the three hulls: empirical tests based on real-world and artificial instances

In this part, datasets from ScotRail will be tested with respect to the number of points in $W',\underline{W}$ and $\overline{W}$. ScotRail is the major passenger rail operator in Scotland and Southern Railway is the major passenger rail operator in South England whose datasets were also used in the experiments to be reported later. Originally, although ScotRail has a train unit fleet with 10 unit types, what makes the scenario quite simple is the fact that for each train service (represented by a node in the network) no more than 4 unit types (commodities) are permitted to cover it. Moreover, there are type coupling compatibility relations that divide the type set into 6 subsets (known as “families” in [14, 15]) of compatible types. This makes computing the convex hulls for any train in the timetable a trivial matter in spaces of dimensions no more than 4 with the number of points less than 9. Tables 3 and 4 give the fleet and coupling upper bound information of ScotRail and Southern Railway.

**Table 3:** Train unit types and their associated families of the fleets of ScotRail and Southern Railway (“c156” stands for “Class 156” and so on)

Operator	Family	Type	Capacity	# of car per unit
			(seats)	
ScotRail	SR.I	c156	145	2
	SR.II	c158	136	2
		c170	189	3
		c170S	198	3
	SR.III	c314	212	3
	SR.IV	c318	219	3
		c320	230	3
	SR.V	c334	183	3
	SR.VI	c380/0	208	3
		c380/1	282	4
Southern Railway	SN.I	c171/7	107	2
		c171/8	241	4
	SN.II	c455/8	316	4
		c456/0	152	2
	SN.III	c313/1	194	3
	SN.IV	c460/0	366	8
	SN.V	c442/1	320	5
	SR.VI	c377/1	223	4
		c377/2	223	4
		c377/3	160	3
		c377/4	243	4

**Table 4:** Coupling upper bounds in number of cars associated with type combinations, regardless of other factors as routes etc., from fleet of ScotRail and Southern Railways

Operator	Family	Combination	Upper bound
			(in # of cars)
ScotRail	SR.I	c156	6
	SR.II	Any combinations by c158, c170, c170S	6
	SR.III	c314	6
	SR.IV	Any combinations by c318, c320	6
	SR.V	c334	8
	SR.VI	Any combinations by c380/0,1	7
Southern Railway	SN.I	c171/7 only	4
		c171/8 only	8
		c171/7 and c171/8	6
	SN.II	c455/8 only	8
		c456/0 only	6
		c455/8 and c456/0	8
	SN.III	c313/1	3
	SN.IV	c460/0	8
	SN.V	c442/1	10
	SN.VI	Any combinations by c377/1,2,3,4	12

We would like to see the potential of the local convex hull method regarding the number of points in the main, up and down hulls on an artificial train with modified type-route and type-type relations that are rendered more complex and difficult. Two kinds of conditions were tested, as:

(i) Assume each train can be served by all the 10 types, but retain the original compatibility relations among unit types and the combination-specific upper bounds.
(ii) Assume each train can be served by all the 10 types, where all unit types are compatible and have the same coupling upper bound.

The first condition makes the convex hull computation into ${\mathbb {R}}_+^{10}$ with a slightly increased number of points. The second condition will give a greatly increased number of points in ${\mathbb {R}}_+^{10}$, where as there are many new combinations that do not exist in practice, unified uppers bound *u* will be used for all combinations varying from 4 to 8 cars which sufficiently covers the real-world cases in ScotRail.

Table 7 in the Appendix gives the exact number of points in the upper ($\overline{W}$), main ($W'$) and down ($\underline{W}$) unit combination sets as triplets $(|\overline{W}|,|W'|,|\underline{W}|)$ for condition (i). When the main set $W'$ cannot be defined since the “single-commodity-presence” condition (16) is not satisfied (usually due to the demand is too high), the number of all points in *W* will be reported as a singleton |*W*|. This reporting style regarding “single-commodity-presence” also applies to Tables 8 and 9 in the Appendix. The passenger demand *r* varies from 25 to 500, which represents the range commonly seen in the ScotRail datasets. Table 8 in the Appendix shows the result based on Condition (ii). In these artificially created scenarios, when the coupling upper bounds are small and the demands are large, no feasible combination can be made, as shown by those cells in the bottom-left corner of the table.

Experiments on the number of points in $\overline{W},W',\underline{W}$ were also performed based on instances from other available sources. Table 9 in the Appendix gives the results on the number of points in *W* taken the Instance A from Cacchiani et al. [6] with 8 train unit types. 50 random number from a uniform distribution on the range of [360, 1404] (as given in [6] as the passenger demand range) were used as the passenger demands. Since in [6] the coupling upper bound is measured in the number of units and for all trains this upper bound is 2 units, it was set that $v_k=1$ and $u=2$ for all the 8 types. Moreover, the 8 types are all compatible.

From the above experiments, for the instances within the context of train unit scheduling, generally the numbers of points in the train combination sets are very small and are thus within the capability of a standard convex hull algorithm. The next section will further justify this conclusion empirically. As for the numbers of outside points in $\overline{W}$ and $\underline{W}$, they can be either small or large compared with the main part $W'$. However, in the real-world example “Instance A” from Cacchiani et al. [6] and with the artificial condition (i) where unit type compatibility is retained, which are the two most realistic instances, the proportions of outside points are all quite small if the three parts can be divided.

### Computing local convex hulls using standard QuickHull under rolling stock scheduling contexts

Some train unit operators may have more difficult problem instances than those tackled by us, and the advancements of railway infrastructure and engineering technology may pose new challenges that might also make the TUSP more complex. Bearing in mind that the local convex hull method cannot be used universally for all kinds of integer multicommodity flow problems, it is useful to have a preliminary idea on its practical range subject to different scenarios. Therefore, three groups of experiments on computing local convex hulls under the context of rolling stock scheduling were conducted. The convex hull computation tool used was an official version of the QuickHull algorithm available from [24]. All experiments were performed on a Dell workstation with 8G RAM and an Intel Xeon E31225 CPU.

The first group of experiments was based on the three instances (A,B,C) from Cacchiani et al. [6], where the authors have analytically proved the inequalities representing relevant local convex hulls for each train and have applied them to their model (also see [10] for more details on the proof). The passenger demand ranges for the three instances are given in $r_A,r_B,r_C$ respectively. In Instance A, there are 8 compatible types with their capacities given in $q_A$. In Instances B and C, there are 10 compatible types respectively with their capacities given in $q_B$ and $q_C$. The coupling upper bound is measured in number of units and is 2 for all trains. The experiments were carried out by increasing the passenger number evenly within the given range and calculating the number of points in the corresponding train unit combinations sets, as shown in Table 10 in the Appendix. The samples with a passenger demand in italic were the ones with the largest number of points in their unit combination sets and having their local convex hull computed by QuickHull by directly taking the points in *W*. Notably as for the three samples from Table 10, since the number of points were too small, the computation times were all displayed as 0’s.

The second group was based on the ScotRail fleet as described before. Originally there were strict type compatibility relation among the 10 types and for each train no more than 4 types can be used. This real-world scenario has no challenge for testing the workability of the local convex hull method. Therefore, an artificial scenario is designed to increase the difficulty in computing the local convex hulls. We assume that all the 10 types are compatible, and for the train to be tested, all the 10 types can be used to serve it. Moreover, we have widened the range of coupling upper bounds to be [4, 12] and the range of passenger demand numbers to be [25, 900]. Table 11 in the Appendix gives the corresponding results. When the passenger demand is too high and the coupling upper bound is too small, there will be no feasible combination (marked as “–”). The maximum number of points in *W* is 1029 in three cells, which are still too easy for QuickHull to compute, by merely 0.006 s.

The third group was based on the datasets from Southern Railway, whose real-world conditions are not challenging for the standard QuickHull algorithm. A similar series of experiments were carried out for the 11 unit types in the Southern Railway fleet, assuming that they were all compatible and were all allowed to serve any trains. The ranges of passenger demand *r* and coupling upper bound *u* were also widened. Table 12 in the Appendix gives the corresponding results. It has some similar patterns as the experiments for ScotRail in Table 11. The convex hull of one of the *u*, *r* pairs yielding the maximum number of combination points, $r=25,u=12$ with 505 points was computed by standard QuickHull, giving a computation time of 0.015 s.

### Computing local convex hulls using standard and 2-facet QuickHull for more difficult cases

Computing local convex hulls in spaces with even higher dimensions (e.g. ${\mathbb {R}}^n_+$ with $n>11$) may not be a real-world issue for most rolling stock scheduling instances. Nevertheless, more difficult conditions may occur in other integer multicommodity problems. Testing more difficult instances will also give useful information on the feasibility range of the local convex hull method. Moreover, a comparison between standard QuickHull and “2-facet” QuickHull can be made under these more difficult conditions because as shown in Sect. 4.3, the instances with $n \le 11$ are not challenging enough.

There are two groups of experiments to be reported in this section. First, a series of experiments on artificial problem instances with a fixed shared coupling upper bounds $u=9$ while varying dimensions *n* from 2 to 21 will be reported. The second group of experiments are based on the same artificial instances with a fixed dimension $n=15$ while varying shared coupling upper bounds *u* from 4 to 12. Both of them are designed to get a better understanding of the behaviors of local convex hull computations in more difficult scenarios. As the dimensions get higher and/or the numbers of points increases due to larger upper bounds, the difficulty in computing relevant local convex hulls often drastically increase. To some certain limits, both the standard and the 2-facet QuickHull will be unable to compute relevant local convex hulls within reasonable time and resources.

#### Varying dimension *n* with fixed coupling upper bound $u=9$

The first group of experiments was conducted on the same machine as described in Sect. 4.3. The instances are artificially created based on the fleets of ScotRail (10 types) and Southern Railway (11 types) by gradually increasing the number of types (assuming all compatible) one by one in the “merged” artificial fleet from 2 to 21 in a way that the types of ScotRail will be included first and the types of Southern Railway will be added later when all the 10 types from ScotRail have been added. A fixed demand $r=107$ and a fixed shared coupling upper bound $u=9$ regardless of type combinations are used. Since 107 is the smallest unit capacity among all unit types, this may give different unit combinations as many as possible. Moreover, since any type can handle the demand 107 on its own, we have $a_i=1, \forall i=1,\ldots ,n$, and this implies $\underline{W}_i=\emptyset , \forall i=1,\ldots ,n$ such that there is no need to compute any down hulls.

Table 5 gives the results of the above experiments. The first column gives the dimensions *n*. The second column gives the number of input points *m* (|*W*| for standard and $|\overline{V}'|+|\overline{W}|$ for 2-facet). The third column gives the number of facets of the computed up hulls including $(b^{-1})^T w=1$. Note that the number of facets of the main hulls will have exactly *n* more facets than the corresponding up hulls. The fourth to seventh columns give the information on the standard QuickHull method in the numbers of processed points *p*, the theoretical average time complexity $mp^{\lfloor n/2 \rfloor -1}$, the numbers of merged facets and the computational times in seconds respectively. The eighth to eleventh columns give the above information on the 2-facet QuickHull method. The computational times in bold indicate the instances where the 2-facet variant performed the same or better than the standard QuickHull.

**Table 5:** Results in computing ${\mathcal {H}}$ by standard and 2-facet QuickHull in ${\mathbb {R}}_+^n$, $n=1...21,u=9,r=107$

*n*	*m*	Up facet #	*p*	$mp^{\lfloor n/2 \rfloor -1}$	# of merged facets	Time (s)	*p*	$mp^{\lfloor n/2 \rfloor -1}$	# of merged facets	Time (s)
	$(|W|,|\overline{V}'|+|\overline{W}|)$		(standard)	(standard)	(standard)	(standard)	(2-facet)	(2-facet)	(2-facet)	(2-facet)
2	14, –	–	4	$m \log m \approx 37$	0	**0**	NN$^{\mathrm{a}}$	NN	NN	NN
3	28,7	5	8	$m \log m \approx 93$	6	**0**	5	$m \log m \approx 14$	1	**0**
4	47,12	7	13	611	24	**0**	8	96	9	**0**
5	72,17	8	16	1152	46	**0**	11	187	26	**0**
6	104,22	9	20	41,600	84	**0**	14	4312	54	**0**
7	144,27	10	26	97,344	171	**0**	17	7803	96	**0**
8	193,32	11	29	4,707,077	238	**0**	22	340,736	200	**0**
9	252,37	12	41	17,368,092	566	**0**	23	450,179	239	**0**
10	280,42	14	57	2,955,680,280	2349	**0.031**	42	130,691,232	1769	**0**
11	353,59	15	64	5,922,357,248	4202	**0.031**	44	221,137,664	2380	**0.015**
12	385,76	16	96	3.13918$\times 10^{12}$	13,860	**0.171**	61	64,189,318,876	10911	**0.124**
13	418,93	17	96	3.40826$\times 10^{12}$	39,141	**1.123**	77	2.51731$\times 10^{12}$	33,266	**0.811**
14	590,166	18	138	4.07499$\times 10^{15}$	265,866	**43.76**	127	6.96515$\times 10^{14}$	262,611	**39.19**
15	703,186	19	161	1.22436$\times 10^{16}$	526,220	165.3	147	1.8768$\times 10^{15}$	542,255	282.9
16	704,187	20	207	1.14648$\times 10^{19}$	1,097,352	**980**	151	3.34719$\times 10^{17}$	643,013	**300.5**
17	704,187	F$^{\mathrm{b}}$	F	F	F	F	171	8.9355$\times 10^{17}$	1,043,420	**1226**
18	777,238	F	F	F	F	F	208	8.33842$\times 10^{20}$	1,043,420	**3,731,451**
19	829,267	F	F	F	F	F	F	F	F	F
20	965,294	F	F	F	F	F	F	F	F	F
21	1022,326	F	F	F	F	F	F	F	F	F

The bold values are the runs where the “2-facet QuichHull” method gave less computational time than the “standard QuickHull” method

$^{\mathrm{a}}$ NN: No need to compute since $\overline{W}=\emptyset$, ${\mathcal {H}}$ is given by $(a^{-1})^Tw \ge 1$ and $(b^{-1})^Tw \le 1$ directly.

$^{\mathrm{b}}$ F: Failed due to various reasons. The most common one was reportedly as “QH6082 qhull error (qh_memalloc)” meaning insufficient memory to allocate relevant data

From Table 5, it can be observed that when $n \le 13$, both methods can compute the local convex hulls very quickly in about 1 s or much less. The time consumptions begin to increase drastically for both methods when $n \ge 14$ and the standard QuickHull failed for $n=17$ up to 21 while the 2-facet QuickHull failed for $n=19$ up to 21. Note that the numbers of up facets as shown in the third column increase almost linearly as the dimensions get larger; however the numbers of merged facets increase in an accelerated manner. As for the numbers of processed points *p*, which have more impact on the time complexity than the total number of points *m*, notice that the proportions of processed points among all (*p* / *m*) for the standard QuickHull are almost always smaller than in 2-facet QuickHull, as the entire hulls generally will contain more interior points. The time complexity columns clearly show how the problem difficulty is drastically increased when the dimension *n* gets larger. In conclusion, the tested instances show that there can be computational limits for the local convex hull method when the dimension and the corresponding theoretical time complexity are large enough, as shown by the last few rows in Table 5.

To compare the two methods, the results obtained from the 2-facet QuickHull are generally better than the standard version, both in theoretical time complexity and actual computational times. Especially when *n* is relatively large as 17 and 18, the standard QuickHull failed while the 2-facet version was still capable of computing the convex hulls. In conclusion, the above results in Table 5 show that the 2-facet QuickHull based on a modification of the standard QuickHull can outperform the latter under some circumstances such as the tested instances.

#### Varying coupling upper bound *u* with fixed dimension $n=15$

**Table 6:** Results in computing ${\mathcal {H}}$ by standard and 2-facet QuickHull in ${\mathbb {R}}_+^{15}$, $u=4...13,r=107$

*n*	*m*	Up facet #	*p*	$mp^{\lfloor n/2 \rfloor -1}$	# of merged facets	Time (s)	*p*	$mp^{\lfloor n/2 \rfloor -1}$	# of merged facets	Time (s)
	$(|W|,|\overline{V}'|+|\overline{W}|)$		(standard)	(standard)	(standard)	(standard)	(2-facet)	(2-facet)	(2-facet)	(2-facet)
4	21,–	–	18	$7.14256 \times 10^8$	27	**0**	NN$^{\mathrm{a}}$	NN	NN	NN
5	48,42	3	45	$3.98581\times 10^{11}$	2604	**0.015**	42	$2.30539\times 10^{11}$	96	**0.015**
6	112,24	3	39	$3.94099\times 10^{11}$	2013	**0.015**	24	$4.58647 \times 10^{9}$	187	**0**
7	193,105	4	93	$1.24869 \times 10^{14}$	122,892	**11.03**	81	$2.96551\times 10^{13}$	4312	**8.377**
8	367,150	3	103	$4.38217\times 10^{14}$	260,435	**85.38**	44	$1.08845\times 10^{12}$	7803	**0.016**
9	703,186	19	161	1.22436$\times 10^{16}$	526,220	165.3	147	1.8768$\times 10^{15}$	542255	282.9
10	1177,549	4	317	$1.19435 \times 10^{18}$	6,040,468	$\mathbf {2.447 \times 10^{4}}$	204	$3.95688 \times 10^{16}$	340,736	$\mathbf {1.57 \times 10^{4}}$
11	2023,1395	F$^{\mathrm{b}}$	F	F	F	F	F	F	F	F
12	3492,–	–	42	$1.91677 \times 10^{13}$	3317	**0.078**	NN	NN	NN	NN
13	5598,2121	F	F	F	F	F	F	F	F	F

The bold values are the runs where the “2-facet QuichHull” method gave less computational time than the “standard QuickHull” method

$^{\mathrm{a}}$ NN: No need to compute since $\overline{W}=\emptyset$, ${\mathcal {H}}$ is given by $(a^{-1})^Tw \ge 1$ and $(b^{-1})^Tw \le 1$ directly.

$^{\mathrm{b}}$ F: Failed due to various reasons. The most common one was reportedly as “QH6082 qhull error (qh_memalloc)” meaning insufficient memory to allocate relevant data

The second group of experiments was conducted based on the same artificial instances as in Sect. 4.4.1. Similarly for the reason to have as many unit combinations as possible, a fixed passenger demand $r=107$ was used for all experiments. As aforementioned, the down hulls will be always empty and the experiments would only consider the up hulls. In addition, a fixed dimension $n=15$ was used which would represent moderate difficult level in terms of dimension as observed from Table 5. The coupling upper bound *u* was varied from 4 to 13 cars. The main purpose of this group is to explore the limit of using the local convex hull method subject to larger numbers of input points as a result of higher coupling upper bounds. It will also make useful comparisons between the standard QuickHull and its 2-facet variant.

Table 6 gives the results of the above experiments. It has the same structure as Table 5 except the first column shows the values of the upper bounds *u*. The computational times in bold indicate the instances where the 2-facet variant used the same or less time than the standard QuickHull. In the two cases of $u=4$ and $u=12$, the up unit combination sets $\overline{W}= \emptyset$ such that the entire convex hulls ${\mathcal {H}}$ are given directly by $(a^{-1})^T w \ge 1$ and $(b^{-1})^T w \le 1$ without any computation if the 2-facet method is used. Note that when $u=12$, it is a multiple of the car numbers of all unit types (either 2, 3 or 4), and by Proposition 3 it is true that $\overline{W}=\emptyset$.

It can be observed that the actual numbers of the facets of the up hulls are still very small, similar as in Table 5. Both the two methods can compute relevant convex hulls very quickly when $u<10$, and the 2-facet method performs better than the standard one. The results for $n=9$ is an exception where the 2-facet performs worse. When $n \ge 10$, the difficulty in convex hull computation increased drastically for both methods, except for $u=12$ which is easy for the standard method and does not need explicit computation by the 2-facet method as ${\mathcal {H}}={\mathcal {H}}'$. This different behavior for $u=12$ is consistent with relevant indicators such as the numbers of processed points, the numbers of merged facets and the theoretical complexity, and is likely a result of the simpler structure of the convex hull making the computation process a lot easier. In all the cases, as long as the convex hulls can be computed, the 2-facet variant generally performs better than the standard version. Moreover, this group of experiments shows there is a computational limit for both methods when the number of points gets larger as given in the last few rows of Table 6.

## Conclusions and future research

In this paper we have generalized the local convex hull method arising in rolling stock scheduling problems using integer multicommodity flow models where each timetabled train is represented by a node in the network graph. Its major characteristic is to use enumeration to capture all the possible combinations at a node with different commodities and the convex hull of such combinations will be computed explicitly if possible. With the nonzero facets of the local convex hulls, a conversion makes the combination variables (based on flow amount per node per commodity) to the original network flow variables (e.g. based on paths) and thus the facets will take their effects as valid inequalities for the original problem. This method can be used to strengthen the LP relaxation as well as to satisfy complex and difficult restrictions such as combination-specific coupling upper bounds. We are especially interested in the feasibility range of this method subject to higher dimensional cases and efficient methods in computing local convex hulls taking advantage of their structures.

We have shown that if the “single-commodity-presence” condition is met, which is a prevailing case in real-world rolling stock scheduling, a local convex hull can be divided into a main hull, an up hull and a down hull, and the main hull can have no more than 2 nonzero facets that are known analytically. For these instances, a 2-facet QuickHull method based on the standard version [3] can be used to compute convex hulls only focusing on outside points. On the other hand, it is also possible that no such three-hull division can be made since some unit types (commodities) cannot be used alone, especially when the passenger demands get large while the coupling upper bound is low. For them the 2-facet QuickHull cannot be used. Nevertheless, in many of such cases the numbers of feasible combinations also tend to be small, which may ease the process of convex hull computation.

Computational experiments on the number of points and computational feasibility for the local convex hull method are reported. As long as the context is set within train unit scheduling with the parameter settings either the same or modified to increase the computational difficulty, the local convex hulls can be easily computed by the standard QuickHull within a very short time. Note that in the UK and other countries where train units are commonly used in passenger railway, it is very rare for a train operator to have a route or train service that can be served by more than 10 types of unit being all compatible with each other. Due to this reason, we find the standard QuickHull sufficient for computing local convex hulls arising in train unit scheduling problems in all cases.

In practice, using the customized 2-facet QuickHull to speed up the computation process is less necessary for train unit scheduling problems. Nevertheless, there might be other fields employing integer multicommodity flow models where the local convex hull method can also be applied. It is possible that some of them may have a far larger number of commodities and combination points at a node or an arc such that the standard QuickHull algorithm may fail or may be less efficient. In those cases, the proposed 2-facet QuickHull may be helpful in improving the computational efficiency or the computability. Section 4.4 gives some computational results on the above point, where there is evidence showing that the 2-facet QuickHull can outperform the standard QuickHull in both the efficiency and computability aspects for the instances tested. When the number of commodity types is even higher (e.g. $>$19) or the number of input points gets larger (e.g. $>$1200) due to higher upper bounds, both the standard and the 2-facet QuickHull will fail in computing the local convex hulls in the tested instances.

The future work on the local convex hull method will be focused on the following aspects. First, more rigorous theoretical investigation on the polyhedral combinatorics side will be conducted. More computational experiments will be carried out on the customized 2-facet QuickHull on other integer multicommodity flow instances. Finally we would like to further design an alternative convex hull computation method by computing sub-hulls with respect to subsets of compatible commodities and then using a final “wrapping” over all sub-hulls to get the entire hull.

## Appendix: Experiment results for Sects. 4.2 and 4.3

See Tables 7, 8, 9, 10, 11, and 12.

**Table 7:** Condition (i) in ${\mathbb {R}}_+^{10}$, passenger demands *r* varies from 25 to 500

*r*	25	50	100	150	200	250	300	350	400	450	500
$(|\overline{W}|,|W'|,|\underline{W}|)$ or |*W*|	(1,25,0)	(1,25,0)	(1,25,0)	(1,23,0)	(1,20,0)	(1,16,0)	14	12	8	2	0

**Table 8:** Numbers of point as “(up, main, down)” or “total” under Condition (ii) in ${\mathbb {R}}_+^{10}$, unified upper bounds $u=4,\ldots ,8$, demand $r=25,\ldots ,500$

$r \backslash u$	4	5	6	7	8
25	(0,13,0)	(14,13,0)	(2,59,0)	(30,59,0)	(56,98,0)
50	(0,13,0)	(14,13,0)	(2,59,0)	(30,59,0)	(56,98,0)
100	(0,13,0)	(14,13,0)	(2,59,0)	(30,59,0)	(56,98,0)
150	(0,11,0)	(14,11,0)	(2,57,0)	(30,57,0)	(56,96,0)
200	8	22	(2,54,0)	(30,54,0)	(56,93,0)
250	4	18	(2,50,0)	(30,50,0)	(56,89,0)
300	–	–	48	76	(56,69,16)
350	–	–	40	68	(56,69,8)
400	–	–	25	53	118
450	–	–	1	29	94
500	–	–	–	–	73

**Table 9:** Numbers of points as “(up, main, down)” or “total” in ${\mathbb {R}}_+^8$ based on “Instance A” from Cacchiani et al. [6]

*r*	1133	625	815	1296	794	1286	1047	1017	395	936
|*W*|	26	(0,40,0)	37	20	37	20	29	32	(0,43,0)	34
*r*	1017	1047	1390	746	804	828	809	774	943	546
|*W*|	29	29	18	38	37	37	37	38	34	(0,40,0)
*r*	1344	389	964	1137	786	389	1065	1285	1085	1360
|*W*|	18	(0,43,0)	34	26	38	(0,43,0)	27	20	27	18
*r*	1056	1105	1373	965	1295	852	1169	621	641	575
|*W*|	29	29	18	34	20	37	24	(0,40,0)	(0,40,0)	(0,40,0)
*r*	1097	424	663	383	1031	837	421	427	659	709
|*W*|	26	(0,43,0)	(0,40,0)	(0,43,0)	32	37	(0,43,0)	(0,43,0)	(0,40,0)	(0,39,0)

Input specifications: $$r \in {\mathcal {U}}[360,1404]$$, $$q \in \{1150,1044,786,702,543,516,495,360\}$$

**Table 10:** Number of combination points based on “Instance A,B,C” from Cacchiani et al. [6]

$r_A$	*360* $^{\mathrm{a}}$	410	460	510	560	610	660	710	760	810	860	910	960	1010	1160	1210	1260	1310	1360	1410
|*W*|	44	43	43	42	40	40	40	39	38	37	36	34	34	33	28	26	24	23	21	19
$r_B$	*590* $^{\mathrm{b}}$	640	690	740	790	840	890	940	990	1040	1090	1140	1190	1240	1290	1340	1390	1440	1490	1530
|*W*|	64	64	64	64	64	61	59	59	58	58	58	57	56	56	56	56	56	52	50	50
$r_C$	*590* $^{\mathrm{c}}$	640	690	740	790	840	890	940	990	1040	1090	1140	1190	1240	1290	1340	1390	1140	1490	1540
|*W*|	64	64	64	64	64	61	59	59	59	59	59	58	57	57	57	57	57	53	51	51

Input specifications: $$r_A \in [360,1404], q_A \in \{1150,1044,786,702,543,516,495,360\}$$, $$r_B \in [588,1534], q_B \in \{1534,1473,1128,980,887,840,834,824,805,588\}$$, $$r_C \in {[}588,1610], q_C \in \{1644,1624,1473,1128,887,840,834,824,805,588\}$$

$^{\mathrm{a}}$ For Instance A, the time to compute the convex hull of the 44 points from $r=360$ by standard QuickHull is displayed as 0 s

$^{\mathrm{b}}$ For Instance B, the time to compute the convex hull of the 64 points from $r=590$ by standard QuickHull is displayed as 0 s

$^{\mathrm{c}}$ For Instance C, the time to compute the convex hull of the 64 points from $r=590$ by standard QuickHull is displayed as 0 s

**Table 11:** Number of points for the artificial instance of ScotRail in ${\mathbb {R}}_+^{10}$

$u \setminus r$	25	50	100	150	200	250	300	350	400	450	500	550	600	650	700	750	800	850	900
4	13	13	13	11	8	4	–	–	–	–	–	–	–	–	–	–	–	–	–
5	27	27	27	25	22	18	–	–	–	–	–	–	–	–	–	–	–	–	–
6	61	61	61	59	56	52	48	40	25	1	–	–	–	–	–	–	–	–	–
7	89	89	89	87	84	80	76	68	53	29	–	–	–	–	–	–	–	–	–
8	154	154	154	152	149	145	141	133	118	94	73	38	–	–	–	–	–	–	–
9	280	280	280	278	275	271	267	259	244	220	199	163	100	16	–	–	–	–	–
10	404	404	404	402	399	395	391	383	368	344	323	287	224	136	51	–	–	–	–
11	635	635	635	633	630	626	622	614	599	575	554	518	455	367	278	–	–	–	–
12	1029	1029	1029$^{\mathrm{a}}$	1027	1024	1020	1016	1008	993	969	948	912	849	761	672	545	335	99	3

$^{\mathrm{a}}$ The time to compute the convex hull of the 1029 points from $r=100,u=12$ by standard QuickHull is 0.006 s

**Table 12:** Number of points for the artificial instance of Southern Railway in ${\mathbb {R}}_+^{11}$

$u \setminus r$	25	50	100	150	200	250	300	350	400	450	500	550	600	650	700	750	800	850	900
4	12	12	12	11	8	3	2	–	–	–	–	–	–	–	–	–	–	–	–
5	17	17	17	16	13	8	6	–	–	–	–	–	–	–	–	–	–	–	–
6	34	34	34	33	30	25	23	12	4	2	–	–	–	–	–	–	–	–	–
7	52	52	52	51	48	43	41	30	19	8	–	–	–	–	–	–	–	–	–
8	96	96	96	95	92	87	85	74	62	44	21	7	3	–	–	–	–	–	–
9	136	136	136	135	132	127	125	114	102	84	57	28	11	–	–	–	–	–	–
10	223	223	223	222	219	214	212	201	189	171	143	107	63	27	9	3	–	–	–
11	320	320	320	319	316	311	309	298	286	268	240	203	154	92	42	15	–	–	–
12	505$^{\mathrm{a}}$	505	505	504	501	496	494	483	471	453	425	388	336	268	178	97	38	15	4

$^{\mathrm{a}}$ The time to compute the convex hull of the 505 points from $r=25,u=12$ by standard QuickHull is 0.015 s

## Footnotes

1 The authors would like to thank an anonymous reviewer for the valuable suggestions on Sect. 2.3.
